# TMTP1-modified nanocarrier boosts cervical cancer immunotherapy by eliciting pyroptosis

**DOI:** 10.7150/thno.108357

**Published:** 2025-04-13

**Authors:** Hanjie Xu, Danya Zhang, Yu Zhang, Yuxin Chen, Yue Sun, Jie Li, Songwei Tan, Ying Zhou, Rui Wei, Fei Li, Ling Xi

**Affiliations:** 1 Department of Obstetrics and Gynecology, National Clinical Research Center for Obstetrics and Gynecology, Tongji Hospital, Tongji Medical College, Huazhong University of Science and Technology, Wuhan, Hubei, 430030, China.; 2 Key Laboratory of Cancer Invasion and Metastasis (Ministry of Education), Hubei Key Laboratory of Tumor Invasion and Metastasis, Tongji Hospital, Tongji Medical College, Huazhong University of Science and Technology, Wuhan, Hubei, 430030, China.; 3 Department of Obstetrics and Gynecology, Centre for Leading Medicine and Advanced Technologies of IHM, The First Affiliated Hospital of USTC, Division of Life Sciences and Medicine, University of Science and Technology of China, Hefei, Anhui, 230001, China.; 4 Department of Obstetrics and Gynecology, Anhui Women and Children's Medical Center, Hefei, Anhui, 230001, China.; 5 School of Pharmacy, Tongji Medical College, Huazhong University of Science and Technology, Wuhan, Hubei, 430030, China.

**Keywords:** pyroptosis, gambogic acid, indocyanine green, nanostructured lipid carrier, tumor immunotherapy

## Abstract

**Rationale:** Pyroptosis, an emerging form of programmed cell death, facilitates the release of tumor antigens and inflammatory factors, which can be leveraged to enhance the efficacy of immune checkpoint blockade (ICB) therapy. However, achieving high-efficiency induction of pyroptosis in cancer cells while minimizing toxicity remains a significant challenge.

**Methods:** In this study, we designed a tumor-targeting peptide TMTP1-modified nanostructured lipid carrier (referred to as TP-NLC) with high loading capacities for gambogic acid (GA) and indocyanine green (ICG). The TMTP1, identified by our research team for its tumor-targeting capabilities, was conjugated to the nanocarrier surface using “click chemistry” to improve the drug delivery efficiency to tumor tissues. The TP-NLC nanocarrier was thoroughly characterized with respect to its morphological attributes, photostability, tumor-targeting capabilities, ability to induce pyroptosis, reactive oxygen species (ROS)-responsive behavior, and anti-tumor efficacy both *in vitro* and *in vivo*.

**Results:** GA encapsulated within the TP-NLC nanocarrier, induced pyroptosis in tumor cells, and enhanced the efficacy of ICG-induced pyroptosis under laser irradiation by disrupting intracellular antioxidant systems, realizing that the combination of GA and ICG synergistically induced caspase-3/GSDME-mediated pyroptosis in a ROS-dependent manner. Tumor cells of pyroptosis released cellular contents and tumor antigens, which subsequently promoted the maturation of dendritic cells (DCs), enhanced intratumoral infiltration of CD8^+^ T cells, initiated systemic antitumor immune response, and augmented the efficiency of PD-1 blockade against both primary and metastatic tumors.

**Conclusion:** The combination of GA and ICG therapy utilizing the constructed nanocarriers presents an attractive therapeutic strategy to trigger pyroptosis and potentiate PD-1 blockade therapy for cervical cancer chemo-immunotherapy.

## Introduction

Despite the implementation of cervical cancer (CC) screening and prophylactic vaccination in many countries and regions, it was estimated that each year 604,127 women are diagnosed with CC and approximately 341,831 succumb to the disease 1. Although early-stage CC can be effectively managed through surgical resection or chemo-radiotherapy, individuals with metastatic, persistent, or recurrent CC experience a poor prognosis. In such cases, platinum-based chemotherapy is primarily palliative, with median overall survival (OS) ranging from 7 to 12 months 2. Despite the administration of the anti-vascular endothelial growth factor (anti-VEGF) agent bevacizumab, the prognosis for patients remains dismal, with a median OS of merely 16.8 months 3. Consequently, there is a pressing need for innovative therapeutic strategies to address the unmet clinical needs of patients with locally advanced or advanced CC. In recent years, tumor immunotherapy targeting the immune checkpoint molecule programmed cell death protein 1 (PD-1) and its ligand, PD-L1, has significantly revolutionized the clinical management of various cancers. Based on the findings of the phase Ⅱ KEYNOTE-158 trial, pembrolizumab, an anti-PD-1 monoclonal antibody, was approved by the United States Food and Drug Administration for the treatment of patients with advanced CC demonstrating PD-L1 expression, following disease progression during or after chemotherapy 4. Although PD-1/PD-L1 blockade has the potential to enhance survival in patients with advanced CC, its clinical application is severely restricted by a low response rate of less than 20% 5. Recent studies have demonstrated that promoting the release of tumor antigens, activating antigen-presenting cells, and increasing T cell infiltration are critical for improving the response rate to PD-1 blockade therapy 6.

Pyroptosis represents a recently identified form of programmed cell death (PCD) that promotes the release of tumor-associated antigens (TAAs) and initiates systemic anti-tumor immunity 7. Proteins from the gasdermin (GSDM) family are considered essential mediators of pyroptosis. These proteins can be cleaved by caspase family proteases, resulting in the generation of the N-terminal fragment of GSDM proteins, which can form pores in the plasma membrane. This pore formation leads to the release of cytoplasmic contents and inflammatory factors, thereby triggering pyroptosis and priming the immune response 8. Recent studies have indicated that elevated levels of reactive oxygen species (ROS) could activate the caspase proteases, which in turn cleave GSDMD or GSDME, thereby triggering pyroptosis 9, 10. Photodynamic therapy (PDT), an innovative treatment modality that relies on photo-induced ROS, has been recognized as a non-invasive cancer therapy characterized by high selectivity, good repeatability, and minimal toxicity 11, 12. Current research has elucidated that PDT could trigger pyroptosis of cancer cells 13. Furthermore, the clinical application of PDT has demonstrated efficacy in the treatment of cervical intraepithelial neoplasia (CIN) 14. Thus, PDT is an extremely potential inducer of pyroptosis in the treatment of CC, offering significant potential for clinical application. Nonetheless, the clinical application of PDT is still restricted by the intracellular antioxidant systems, including the thioredoxin (Trx) and glutathione (GSH) systems 15, 16. Consequently, there is a pressing need to develop innovative strategies for the efficient and simultaneous delivery of antioxidant blockers and photosensitizers into tumors to enhance PDT and effectively induce pyroptosis.

**Scheme 1:**
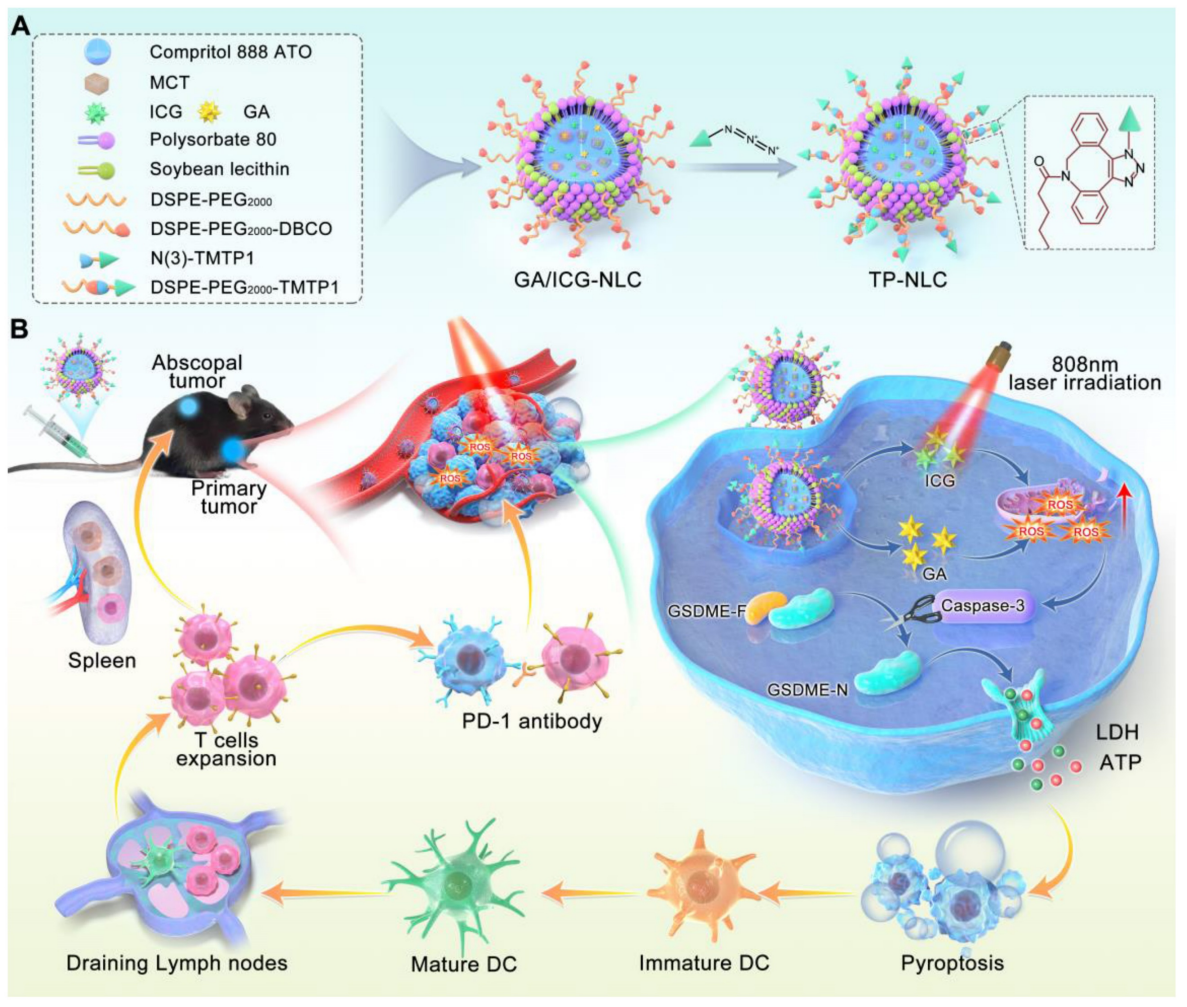
Schematic illustration mechanism of the TP-NLC for cancer immunotherapy by pyroptosis induction and PD-1 blockade. (A) Synthesis of TP-NLC. (B) The mechanism of TP-NLC for boosting PD-1 blockade therapy by inducing GSDME-mediated pyroptosis.

Nanocarrier systems offer an excellent solution for achieving high-efficiency and synchronous delivery, and they have been utilized in tumor immunotherapy 17. Recent studies have reported the application of various nanoparticles for pyroptosis-mediated tumor immunotherapy, focusing on the induction of pyroptosis through diverse strategies, such as delivery of chemotherapeutic agents, precise delivery photosensitizer molecules into mitochondria, mitochondrial Ca^2+^ overload, and ROS storm generated by sonodynamic therapy 18-21. The induction of specific pyroptosis in cancer cells and the dynamic regulation of drug biodistribution were also nano-pyroptosis strategies for cancer therapy, aimed at minimizing systemic side effects 22, 23. Furthermore, to enhance immune system activation, combining pyroptosis with other forms of PCD, such as ferroptosis and cuproptosis, offered a novel nano-pyroptosis strategy 24, 25. These research results indicated that the exploration of nanoplatforms holds significant promise for pyroptosis-mediated cancer treatment. However, the clinical application of nanoplatforms is hindered by challenges such as the uncontrolled induction of pyroptosis by nanoparticles, their slow *in vivo* metabolism, and inefficient delivery to tumor tissues. To improve delivery efficiency, active targeting can be employed. This approach utilizes specific targeting ligand on nanocarriers to bind receptors expressed on target cells, thereby achieving precise spatial localization for the targeted delivery of agents 26. TMTP1, a five-amino peptide (NVVRQ) screened by FliTrx bacterial peptide display system in our laboratory, could specifically target various primary tumors and metastases, and has been shown to increase the cellular uptake of nanocarriers in tumors 27-29. Gambogic acid (GA), a xanthone structure isolated from Gamboge, exerts anticancer, antioxidant, anti-inflammatory, and anti-proliferative effects 30. Notably, our previous research indicated that GA can induce pyroptosis of cancer cells via the caspase-3/GSDME signaling pathway 31. Herein, based on the antioxidant and pyroptosis induction effects of GA, we developed a TMTP1-modified nanostructured lipid carrier (TP-NLC) for co-delivery of GA and indocyanine green (ICG) as a potent pyroptosis inducer to enhance tumor immunogenicity for boosting the efficiency of PD-1 blockade. TP-NLC was spherical structures with a hydrophobic core encapsulating GA and ICG through hydrophobic interactions, and was modified with the TMTP1 peptide using click chemistry reaction between PEGylated dibenzocycloctyne (DBCO) and azido (N(3)) conjugates, thereby improving the capacity of targeting CC tissues *in vivo* (Scheme 1A).

The co-delivery strategy utilizing nanostructured lipid carrier (NLC) not only effectively encapsulated GA and ICG, but also improved circulating half-life of the drugs, addressed the hydrophobicity of GA and the photobleaching of ICG, and amplified ROS-mediated cytotoxicity by inhibiting the intracellular antioxidant systems. Due to the presence of polyethylene glycol (PEG), TP-NLC exhibited high encapsulation efficiency (EE), excellent stability, and remarkable biocompatibility. TP-NLC could selectively reach the tumor tissues and be internalized into tumor cells through the passive targeting of enhanced permeability and retention (EPR) effect and the active targeting of TMTP1 peptide after entering blood circulation. Once internalized, TP-NLC triggered pyroptosis of CC cells through the ROS/caspase-3/GSDME-dependent pathway under 808 nm near-infrared (NIR) laser irradiation, which promoted the maturation of dendritic cells (DCs), facilitated the differentiation and infiltration of CD8^+^ T cells, activated the adaptive immune response, and augmented the efficacy of anti-PD-1 therapies in CC, thus conferring the regression of both primary and metastatic tumors (Scheme 1B).

## Results and Discussion

### Synthesis and characterization of TP-NLC nanocarriers

Uniform and spherical nanostructured lipid carriers (NLC) co-loaded with GA and ICG, as shown in Scheme 1A, were synthesized by the emulsification-solvent evaporation method, leveraging both hydrophilic and hydrophobic interactions. To achieve the tumor-targeting ability and ensure adequate intracellular accumulation of GA/ICG-NLC, the tumor-targeting peptide TMTP1 was effectively conjugated to the surface of the GA/ICG-NLC through a click chemistry reaction. The 1, 2-distearoyl-sn-glycero-3-phosphoethanolamine (DSPE)-polyethylene glycol 2000 (PEG_2000_)-DBCO (DSPE-PEG_2000_-DBCO) was incorporated into GA/ICG-NLC through pre-insertion method (DSPE-PEG_2000_-DBCO was added into the oil phase before synthesis) to obtain PEGylated NLC and provide a strong anchor for binding to TMTP1-N(3) through click chemistry reaction (Scheme 1A). The TMTP1 peptide was successfully modified on the surface of GA/ICG-NLC with a high coupling efficiency of 89.67 ± 3.21%, resulting in the construction of TP-NLC (Figure S1).

**Figure 1:**
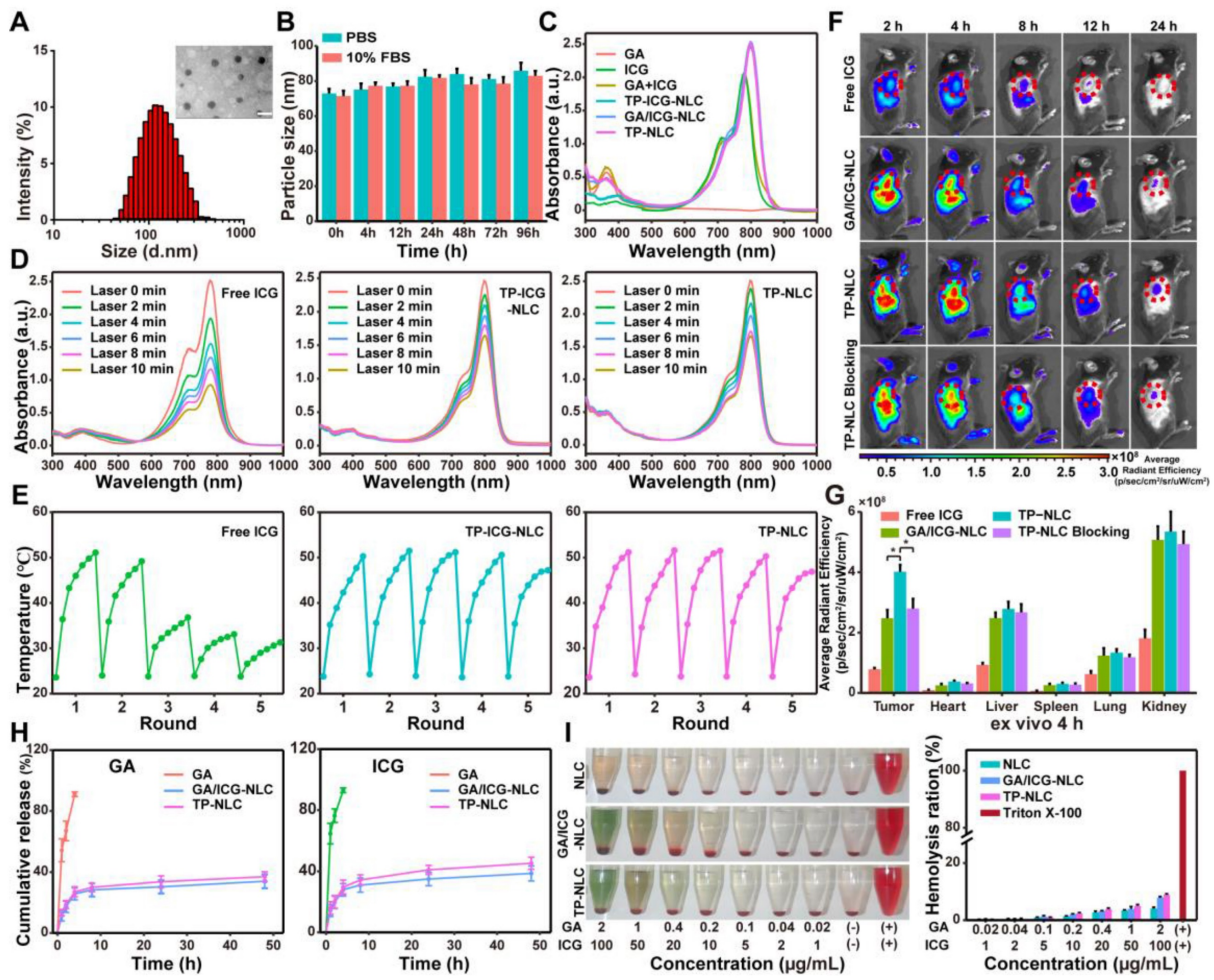
Characterization and body distribution of TP-NLC nanocarrier. (A) Hydrodynamic size distribution and TEM image of TP-NLC nanocarrier. Scale bar: 100 nm. (B) The particle size of TP-NLC in PBS and 10% FBS within 96 h stored at 37 ℃. (C) The UV-vis absorption spectra of different formulations (GA, ICG, GA+ICG, TP-ICG-NLC, GA/ICG-NLC, and TP-NLC). (D) The UV-vis absorption spectra of free ICG, TP-ICG-NLC, and TP-NLC solution irradiated with laser irradiation (808 nm, 1.2 W/cm^2^) for different times. (E) Photothermal stability of free ICG, TP-ICG-NLC, and TP-NLC during five cycles of laser on/off irradiation (808 nm, 2 W/cm^2^). (F) *In vivo* NIR fluorescence images of free ICG, GA/ICG-NLC, and TP-NLC in subcutaneous TC-1 xenograft model mice at 2 h, 4 h, 8 h, 12 h, and 24 h after intravenous administration. In the TP-NLC Blocking group, TMTP1 peptide as competitive inhibitor was pre-injected by tail vein before the administration of TP-NLC. (G) Quantitative analysis of the fluorescence intensity of *ex vivo* tumor tissues and major organs (heart, liver, spleen, lung, and kidney) at 4 h post-injection. (H) The cumulative release of GA and ICG from TP-NLC within 48 h. (I) Hemolysis photographs and hemolysis ratios of different nanocarriers at various GA and ICG concentrations. *: *P* < 0.05.

Transmission electron microscopy (TEM) confirmed the homogeneous and spherical morphology of TP-NLC, with an average diameter of TP-NLC approximately 47 nm, which was smaller than the average hydrated particle size of 72.23 ± 6.35 nm (Figure 1A and S2A). As shown in Figure S2B-D, the GA/ICG-NLC exhibited a morphology comparable to that of TP-NLC, with an average diameter of approximately 44 nm. However, the GA/ICG-NLC demonstrated a slightly reduced hydrodynamic diameter (63.92 ± 5.21 nm) compared to TP-NLC, attributable to the absence of TMTP1 modification. Dynamic light scattering (DLS) analysis revealed that the polydispersity index (PDI) of TP-NLC was 0.25 ± 0.04, with a negative or near-neutral surface zeta potential, a characteristic shared by GA/ICG-NLC. As depicted in Figure 1B and S3, the hydrodynamic diameters of both TP-NLC and GA/ICG-NLC in PBS and 10% fetal bovine serum (FBS) remained relatively unchanged over 96 h, as measured by DLS, indicating their excellent stability. The ultraviolet-visible (UV-vis) spectra, presented in Figure 1C, revealed that both TP-NLC and GA/ICG-NLC exhibited two distinct absorbance peaks: a prominent peak at 800 nm, corresponding to free ICG, and a smaller peak at 361 nm consistent with free GA, indicating the efficient co-encapsulation of GA and ICG. Utilizing the specificity of high-performance liquid chromatography (HPLC) and UV-vis, the standard curves were constructed by plotting the areas under the HPLC peak against GA and UV-vis absorption against ICG, respectively (Figure S4 and S5). Based on these standard curves, the EE of GA and ICG were determined to be 97.79 ± 1.41% and 96.02 ± 2.54% for TP-NLC, and 98.02 ± 1.29% and 96.87 ± 3.12% for GA/ICG-NLC, respectively.

Then, the photostability of TP-NLC in terms of anti-bleaching was investigated. The absorption spectra of free ICG, TP-ICG-NLC, and TP-NLC were recorded under 808 nm NIR laser irradiation at a moderate-intensity (1.2 W/cm^2^) over varying time intervals. As shown in Figure 1D, the absorption of ICG showed a rapid decline within 10 min of laser irradiation exposure, whereas both TP-NLC and TP-ICG-NLC demonstrated a more gradual decrease in absorption, indicating that TP-NLC and TP-ICG-NLC possessed significantly enhanced photostability, primarily attributed to the effective stabilization and reduced degradation of ICG facilitated by the NLC nanocarriers. To further assess the photothermal stability, the light-to-heat conversion efficiency of TP-NLC was evaluated under multiple cycles of 808 nm NIR laser irradiation. Owing to its superior photothermal conversion properties under NIR laser irradiation, ICG has been extensively utilized in PDT and photothermal therapy (PTT). To more accurately assess the photothermal stability of TP-NLC, a high-intensity NIR laser (2 W/cm^2^) was employed. Following five cycles of on/off irradiation, ICG demonstrated markedly diminished photothermal conversion efficiency, attributed to the photobleaching effect. In contrast, both TP-NLC and TP-ICG-NLC retained robust photothermal conversion efficiency, which can be attributed to the enhanced stability of ICG conferred by the well-structured nanoassembly (Figure 1E). Additionally, as depicted in Figure S6, free ICG in aqueous solution appeared yellow with a loss of green coloration after 10 min irradiation, whereas TP-NLC retained its green appearance. Furthermore, the ROS levels of free ICG, GA+ICG, GA/ICG-NLC and TP-NLC+L under 808 nm NIR laser irradiation (0.5 W/cm^2^) for 5 min were assessed. As shown in Figure S7, the ROS levels in the GA/ICG-NLC+L and TP-NLC+L groups were elevated relative to the ICG+L group, with the highest ROS levels being observed in the GA+ICG+L group. However, no significant differences were detected among the combination groups. GA alone did not affect ROS generation at the extracellular levels. The results presented above demonstrated that GA could enhance ROS generation by ICG, while the nanocarrier did not influence ROS generation. These findings suggested that TP-NLC exhibited excellent photostability and was a suitable agent for PDT in cancer.

An effective tumor-targeted delivery system not only enhances the antitumor efficacy by delivering therapeutic agents into tumor cells, but also reduces drug-induced adverse effects by minimizing nonspecific distribution throughout the body 32. Consequently, the biodistribution and tumor targeting ability of TP-NLC were investigated prior to conducting antitumor experiments and were compared with those of free ICG and GA/ICG-NLC. First, the targeting ability of TP-NLC to CC cells *in vitro* was assessed using coumarin 6 (Cou-6) as a tracer. The uptake of TP-Cou-6-NLC, Cou-6-NLC, and free Cou-6 by tumor cells was detected by confocal laser microscopy. In HeLa and TC-1 cells, the TP-Cou-6-NLC group exhibited stronger fluorescence intensity compared to the Cou-6-NLC and free Cou-6 groups, which could be inhibited by an excess of linear peptide TMTP1 (Figure S8). However, the fluorescence intensity of the normal HaCaT cells in the TP-Cou-6-NLC group was comparable to that observed in the Cou-6-NLC group (Figure S9). These results demonstrated that TP-Cou-6-NLC could specifically target CC cells rather than normal cells. Subsequently, the distribution of TP-NLC in tumor-bearing mice was detected. Following intravenous administration of free ICG, GA/ICG-NLC, or TP-NLC, the fluorescence signals from TC-1 tumor-bearing C57BL/6 mice were monitored using an IVIS imaging system at predetermined time points. As shown in Figure 1F, a bright fluorescence signal from TP-NLC was detected at the tumor sites, which gradually increased over the first 4 h post-injection, and subsequently decreased. Notably, the bright fluorescence signal of TP-NLC was significantly inhibited when an excess of linear peptide TMTP1 was administered via the tail vein prior to TP-NLC injection. In contrast, the fluorescence signals of both GA/ICG-NLC and free ICG at the tumor sites were weaker and gradually diminished over time following injection. At 4 h and 24 h post-injection, the mice were euthanized, and their major organs were harvested for *ex vivo* imaging. The signals of free ICG, GA/ICG-NLC, and TP-NLC were predominantly detected in the kidneys, livers, and tumors (Figure S10 and S11). Moreover, at 4 h post-injection, the TP-NLC signal was more pronounced than those of free ICG, GA/ICG-NLC, and TP-NLC with excess peptide TMTP1, indicating that TP-NLC exhibited superior tumor targeting ability and could effectively deliver GA and ICG to tumor tissues (Figure 1G). Then, the release profiles of GA and ICG from the TP-NLC nanocarrier were further evaluated. As presented in Figure 1H, GA was quickly released within 4 h, whereas the accumulative release percentages of GA from GA/ICG-NLC and TP-NLC were approximately 34% and 37% at 48 h, respectively. A similar rapid release of ICG was also observed within 4 h, with the release amount reaching approximately 39% from GA/ICG-NLC and about 45% from TP-NLC at 48 h. The above results demonstrated that both TP-NLC and GA/ICG-NLC could avoid the rapid release of GA and ICG. Additionally, the hematological biocompatibility of TP-NLC carrier was evaluated through hemolysis assays. As demonstrated in Figure 1I, the hemolysis ratios of NLC, GA/ICG-NLC, and TP-NLC nanocarriers were less than 10%, even at the maximum GA and ICG concentrations (2 μg/mL and 100 μg/mL, respectively), indicating that these nanocarriers exhibited favorable hematological biocompatibility and were relatively safe for intravenous injection.

### Pyroptosis analysis of TP-NLC+L triggered

**Figure 2:**
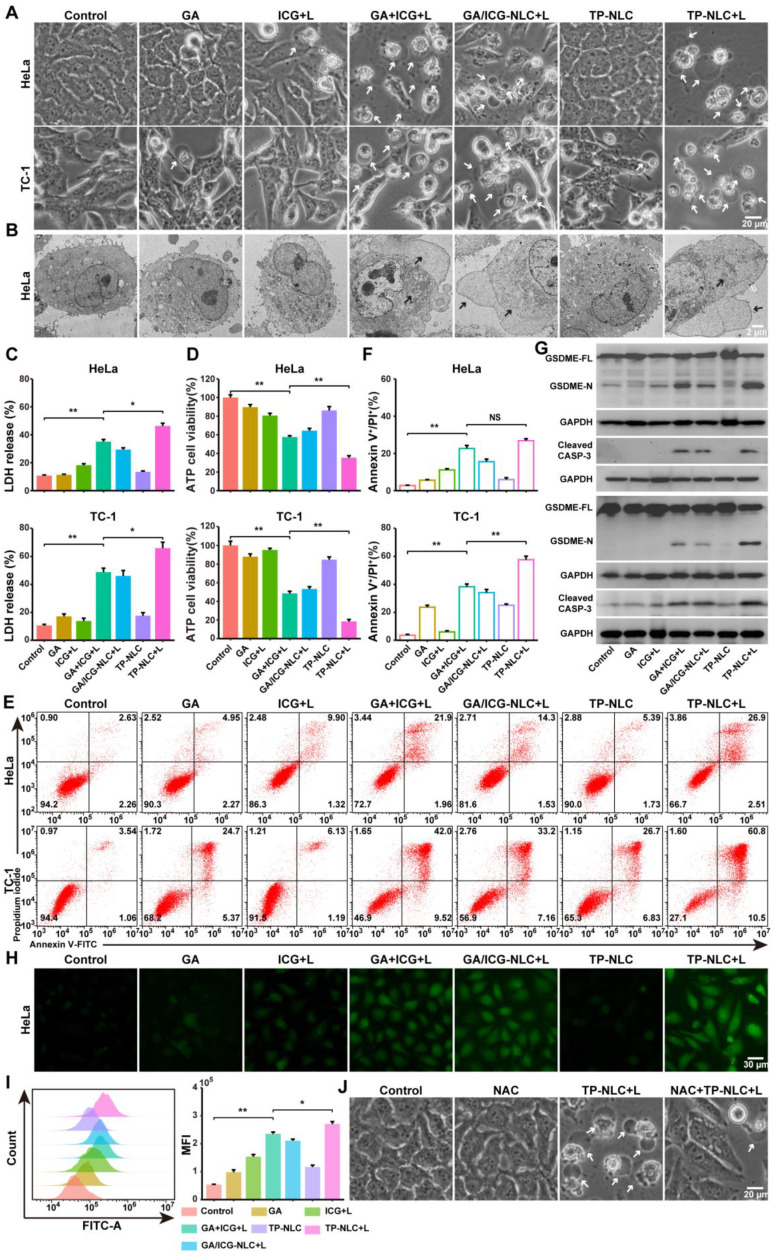
TP-NLC+L triggered cell pyroptosis in CC cells, associated with intracellular ROS accumulation. (A) Representative bright-field images of HeLa and TC-1 cells treated with PBS, free ICG+L, GA, GA+ICG+L, GA/ICG-NLC+L, TP-NLC, and TP-NLC+L. +L: laser irradiation at 808 nm (0.5 W/cm^2^, 5 min). The white arrows indicated pyroptotic cells. Scale bar: 20 μm. (B) The representative TEM images of HeLa cells treated by different treatments. The black arrows indicated the large bubbles and organelle edema. Scale bar: 2 μm. (C) The release of LDH and (D) ATP cell viability after different treatments. (E) Flow cytometric analysis and (F) absolute quantification of HeLa and TC-1 cells after different treatments. (G) Western blot detection of full-length GSDME (GSDME-FL), GSDME-N terminal domain (GSDME-N), and Cleaved CASP-3 expressions in HeLa (upper panel) and TC-1 (lower panel) cells after different treatments. (H) Representative fluorescent images and (I) flow cytometric analysis of ROS generation in HeLa cells after different treatments, detected by the fluorescent dye DCFH-DA. Scale bar: 30 μm. (J) Representative bright-field images of HeLa cells treated with TP-NLC in the presence or absence of NAC. The white arrows indicated pyroptotic cells. Scale bar: 20 μm. *: *P* < 0.05, **: *P* < 0.01.

The induction of pyroptosis is a crucial event for successful antitumor treatment. Consequently, the pyroptosis-inducing abilities of free ICG, GA, GA+ICG, GA/ICG-NLC, and TP-NLC were assessed in HeLa and TC-1 cells. Prior to this evaluation, the cytotoxicity of TP-NLC in HeLa cells was investigated. As described in Figure S12, cell viability was inhibited in a dose-dependent manner. The IC50 values were determined to be 3.31 μg/mL for GA and 59.62 μg/mL for ICG+L treatments. For HeLa cells treated with GA+ICG+L, the IC50 values were 0.79 μg/mL (GA) and 39.31 μg/mL (ICG), while TP-NLC exhibited a relatively low IC50 value. To further explore the potential synergistic therapeutic effect of combining GA and ICG, the combination index (CI50) for treatments was calculated. The results indicated that the CI50 values for GA+ICG+L, GA/ICG-NLC+L, and TP-NLC+L were less than 1, signifying synergistic cytotoxicity. To initiate PDT rather than PTT, a low-intensity NIR laser (0.5 W/cm^2^) was employed to maintain the temperature below 45 ℃ during treatment 33. HeLa and TC-1 cells were incubated with the corresponding formulations for 3 h, followed by the addition of fresh medium for an additional 21 h. Subsequently, pyroptosis-related indicators were assessed. Temperature monitoring during treatment revealed that the maximum temperature in each group did not exceed 30.0°C, indicating the activation of PDT rather than PTT (Figure S13). As shown in Figure 2A, after combined treatment with GA and ICG (+L, with laser irradiation), HeLa and TC-1 cells exhibited morphological characteristics of pyroptosis, such as cell membrane ballooning, which was distinct from the morphological features of apoptosis, characterized by cell membrane shriveling. TEM confirmed the presence of cell membrane ballooning, and intracellular organelle swelling was observed in pyroptotic cells due to the influx of extracellular water through polymeric pores in cellular membranes (Figure 2B). Additionally, to verify the presence of polymeric pores in the cell membrane resulting from pyroptosis, standard experimental methods commonly employed in pyroptosis research, such as cellular adenosine triphosphate (ATP) content measurement, lactate dehydrogenase (LDH) release assays, and the proportions of Annexin V-FITC^+^/PI^+^ cells, were conducted 34. As shown in Figure 2C, the LDH release in the supernatant of cells treated with GA and ICG was higher than that of cells treated alone, and the highest levels of LDH release were observed in the TP-NLC+L group, followed by the GA+ICG and GA/ICG-NLC+L groups. Similarly, the intracellular ATP levels in the TP-NLC+L group were lower than those observed in the other groups (Figure 2D). Furthermore, pyroptosis in HeLa and TC-1 cells was further measured using Annexin V-FITC and propidium iodide (PI) staining. As presented in Figure 2E-F, corresponding to the aforementioned results, the percentages of pyroptotic cells (Annexin V-FITC^+^/PI^+^ cells) in both HeLa and TC-1 cells were higher in the combined drug groups (GA+ICG+L, GA/ICG-NLC+L, and TP-NLC+L) compared to the single drug groups (GA, ICG+L, and TP-NLC) and the control group. In the combined drug groups, the TP-NLC+L group exhibited the highest percentage of pyroptotic cells, followed by the GA+ICG+L group and the GA/ICG-NLC+L group, which was attributed to the superior tumor-targeting properties of TP-NLC and the distinct mechanisms by which the drugs enter the cells: free GA and ICG rapidly diffused into the cells, whereas the GA/ICG-NLC nanocarrier was internalized via an energy-dependent endocytosis process. Then, western blotting assay was conducted to examine the expression of pyroptosis-related proteins in HeLa and TC-1 cells. As presented in Figure S14**,** the cleavage of GSDMD into its N-terminal fragment (GSDMD-N) was not detected in any group. However, the N-terminal fragment of GSDME (GSDME-N) was observed exclusively in the combined drug groups, with the highest expression level in the TP-NLC+L group, followed by the GA+ICG+L group (Figure 2G). Corresponding to these findings, cleaved caspase-3 increased across all the combined drug groups. These comprehensive results demonstrated that TP-NLC effectively induced pyroptosis in CC cells through caspase-3-mediated GSDME cleavage.

ROS was recognized as the primary agents responsible for cell death induced by PDT and have been shown to trigger cell pyroptosis 13, 35. Consequently, the capacity of TP-NLC to generate ROS under NIR laser irradiation was assessed using the 2'7'-dichlorofluorescein diacetate (DCFH-DA) fluorescence probe. As presented in Figure 2H-I and S15-16, the intracellular ROS levels induced by the combined drug groups were significantly higher than those induced by the single drug groups both in HeLa and TC-1 cells. Notably, the TP-NLC+L group exhibited the highest intracellular ROS levels among all the groups. These findings implied that the ability of GA combined with ICG to induce pyroptosis was related to intracellular ROS generation. To further investigate the role of ROS in pyroptosis induced by the GA and ICG combination, N-acetylcysteine (NAC), a known ROS scavenger, was utilized in subsequent experiments. Firstly, the changes in cellular morphology were observed, revealing that the morphological characteristics of pyroptosis induced by TP-NLC+L were mitigated by NAC (Figure 2J and S17A). Additionally, significant decreases in LDH release, triggered by TP-NLC+L, were detected in HeLa and TC-1 cells pretreated with NAC (Figure S17B). Finally, western blotting was conducted, revealing that NAC significantly reduced the production of GSDME-N and cleaved caspase-3 (Figure S17C). Considering that there was a positive feedback loop between ROS and mitochondrial damage, the alterations of mitochondrial membrane potential were assessed using JC-1 dye as a fluorescent indicator. As presented in Figure S18, strong red fluorescence was detected in both the control and single drug groups. In contrast, the combined drug groups exhibited reduced red fluorescence and increased green fluorescence, with the most pronounced red-to-green fluorescence shift occurring in the TP-NLC+L group, indicating that the combination of GA with ICG+L could cause mitochondrial damage, with the TP-NLC+L group experiencing the most significant damage. Collectively, these results indicated that TP-NLC+L induced pyroptosis in both HeLa and TC-1 cells, a process that was dependent on intracellular ROS generation.

### *In vivo* antitumor effect of TP-NLC+L

**Figure 3:**
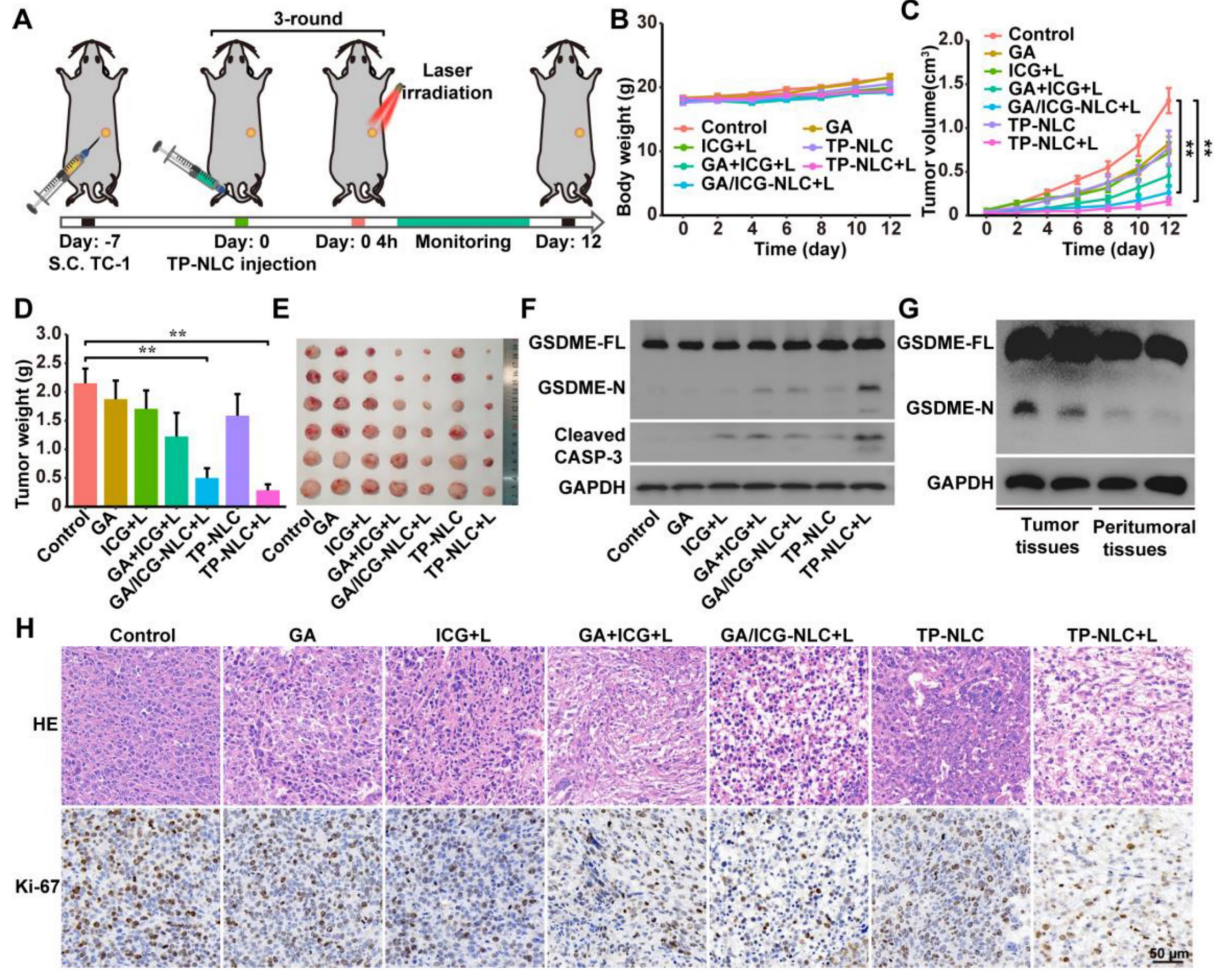
TP-NLC+L triggered pyroptosis for antitumor efficacy *in vivo*. (A) Treatment schedule for TP-NLC+L therapy. (B) Change curves of mice body weight after PBS, free ICG+L, GA, GA+ICG+L, GA/ICG-NLC+L, TP-NLC, and TP-NLC+L treatments. (C) Tumor growth curves, (D) tumor weights, (E) and tumor photographs of isolated tumor tissues after different treatments. (F) Western blot detection of full-length GSDME (GSDME-FL), GSDME-N terminal domain (GSDME-N), and Cleaved CASP-3 expressions in tumor tissues. (G) Western blot detection of GSDME-FL and GSDME-N in tumor tissues and paired peritumoral tissues of TP-NLC+L group. (H) The representative hematoxylin-eosin (HE) and Ki-67 immunohistochemistry staining images of tumors after different treatments. +L: laser irradiation at 808 nm (0.5 W/cm^2^, 10 min). Scale bar: 50 μm. *: *P* < 0.05, **: *P* < 0.01.

Based on the ability of TP-NLC+L to induce pyroptosis and its advantageous tumor-targeting properties, we further evaluated the antitumor efficacy of TP-NLC+L in a TC-1 tumor-bearing mouse model. As illustrated in Figure 3A, the TC-1 tumor-bearing model was established in C57BL/6 mice through subcutaneous injection of 2 × 10^5^ TC-1 cells. Seven days post-injection, the mice were randomly divided into the control, GA, ICG+L, GA+ICG+L, GA/ICG-NLC+L, TP-NLC, and TP-NLC+L groups. The corresponding drugs were injected via the tail vein every three days for a total of three times. Based on the *in vivo* imaging results of TP-NLC, laser irradiation was conducted 4 h following the administration of free ICG, GA/ICG-NLC and TP-NLC (Figure 3A). Tumor volumes and body weights of mice were recorded every two days. As shown in Figure 3B, the treatment groups exhibited significant antitumor effect compared to the control group, with minimal side effects as indicated by stable body weights across all groups. As presented in Figure 3C-E, the groups treated with GA, ICG+L, and TP-NLC exhibited significantly larger volumes and weights of tumors, compared with the GA+ICG+L, GA/ICG-NLC+L and TP-NLC+L groups. Notably, the TP-NLC+L demonstrated the most effective tumor control. Furthermore, the GA/ICG-NLC+L group showed a more pronounced antitumor effect than the GA+ICG+L group, indicating that the NLC-mediated delivery improved the therapeutic efficacy relative to the GA+ICG+L treatment without nanocarrier-mediated delivery.

To investigate whether pyroptosis was involved in the treatment process, the expressions levels of GSDME and cleaved caspase-3 proteins in tumor tissues were analyzed by western blot assays. As shown in Figure 3F, the N-terminal fragments of GSDME were markedly increased in the tumor tissues treated with TP-NLC+L, along with elevated expressions of cleaved caspase-3, confirming the involvement of pyroptosis in the antitumor effect mediated by TP-NLC+L treatment.

To further assess the pyroptosis-inducing selectivity of TP-NLC on tumor cells, the expression levels of GSDME in both tumor tissues and adjacent peritumoral tissues were also detected by western blot assays. As presented in Figure 3G, the presence of GSDME-N was detected in tumor tissues subjected to TP-NLC+L treatment, but not in the corresponding peritumoral tissues. This finding reaffirms the effective tumor-targeting capability of TP-NLC and its selective induction of pyroptosis in tumor cells. Moreover, the more tumor cells suffered structural changes with poor states and the fewer proliferating cells labeled with Ki-67 were observed in the TP-NLC+L group (Figure 3H). These results indicated that TP-NLC+L treatment exerted significant cytotoxic effects on tumor cells by inducing pyroptosis.

### The antitumor immune response initiated by TP-NLC+L

**Figure 4:**
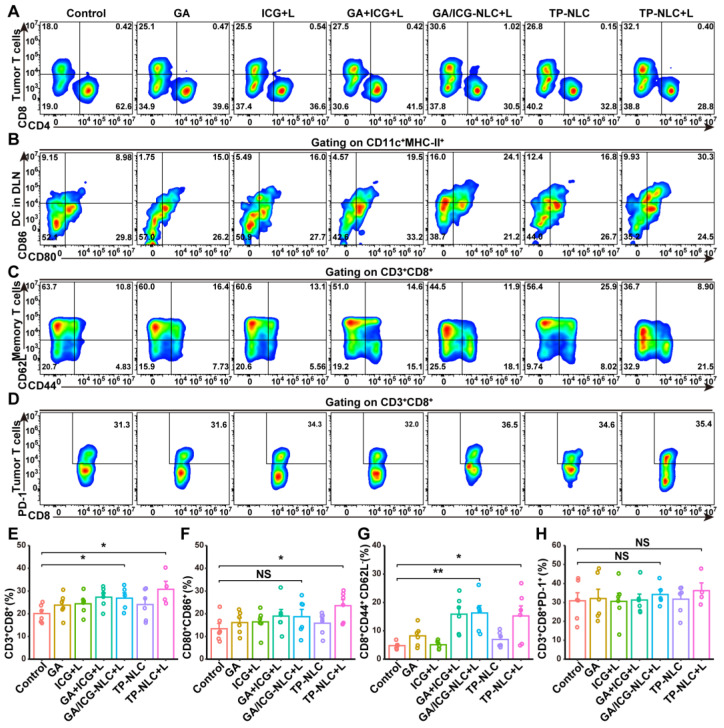
Pyroptosis of tumor cells initiated immune response. (A) Representative flow cytometric analysis and (E) quantitative analysis of CD8^+^ T cells proportion gating on CD3^+^ TILs. (B) Representative flow cytometric analysis and (F) quantitative analysis of CD80^+^CD86^+^ T cells proportion gating on CD11c^+^MHC-II^+^ cells in the TDLN tissues. (C) Representative flow cytometric analysis and (G) quantitative analysis of CD8^+^CD44^+^CD62L^-^ effector memory T cells (T_em_) proportion in the spleen tissues. (D) Representative flow cytometric analysis and (H) quantitative analysis of PD-1^+^ T cells proportion gating on CD3^+^CD8^+^ T cells in the tumor tissues. NS: not significant, *: *P* < 0.05, **: *P* < 0.01.

Recent studies have indicated that the pyroptosis of tumor cells could promote the maturation of DCs and increase the clonal expansion of T cells, thereby contributing to antitumor immune responses 36, 37. To investigate the immune effects *in vivo*, flow cytometric analysis and subsequent quantification were performed to assess the proportion of tumor-infiltrating lymphocytes (TILs). The results demonstrated a significant increase in the presence of CD3^+^CD8^+^ TILs in the TP-NLC+L group (Figure 4A and 4E). Given that tumor-derived DCs are pivotal in antigen uptake and presentation to T cells within the lymph nodes, thereby initiating antitumor immune responses, the maturation of DCs in tumor-draining lymph nodes (TDLN) was further assessed by flow cytometry. As presented in Figure 4B and 4F, the percentage of mature DCs (CD11c^+^MHC-II^+^CD80^+^CD86^+^) was significantly elevated in the TP-NLC+L group, compared with the control group, suggesting that TP-NLC+L treatment effectively promoted DCs maturation. The observed expansion of CD8^+^ T cells clone and DCs maturation indicated that TP-NLC+L treatment could elicit a robust adaptive immune response. The formation of immunological memory is a hallmark of the adaptive immune response. Therefore, CD44 and CD62L expressions on CD8^+^ T cells were measured to detect the central memory T (T_cm_) cells and effector memory T (T_em_) cells. As shown in Figure 4Cand 4G, the percentage of CD8^+^ T_em_ (CD44^+^CD62L^-^) cells in the spleens was increased after GA/ICG-NLC+L or TP-NLC+L therapy, which indicated the formation of protective immune responses. Furthermore, to explore the effects of TP-NLC+L therapy on alleviating CD8^+^ T cells exhaustion, PD-1 expressions on the surface of CD8^+^ TILs were assessed. The analysis revealed no significant change in the percentage of CD8^+^PD-1^+^ T cells in the TP-NLC+L group compared to other groups, as shown in Figure 4D and 4H. These results indicated that cell pyroptosis triggered by TP-NLC+L could induce DC maturation, CD8^+^ T cells clonal expansion, and the initiation of adaptive immune responses.

### TP-NLC+L enhanced the therapeutic efficacy of PD-1 blockade in CC

**Figure 5:**
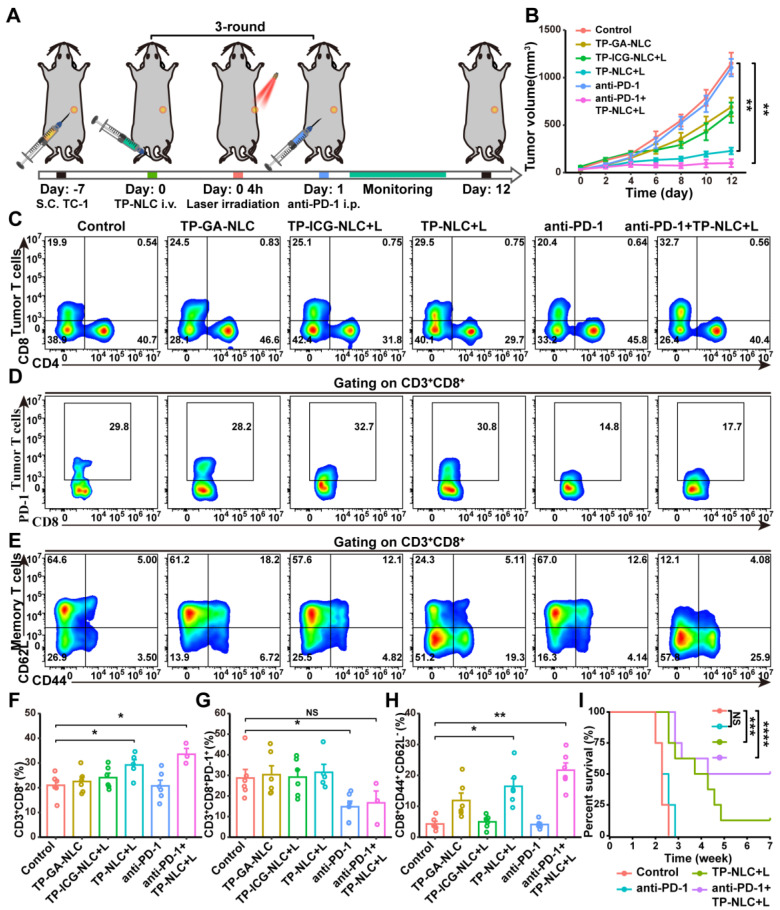
*In vivo* antitumor efficacy and immune response induced by TP-NLC+L in combination with PD-1 blockade therapy. (A) Treatment schedule for the combination of TP-NLC+L and PD-1 blockade therapy. (B) Tumor growth curves of each group (n = 6). (C) Representative flow cytometric analysis and (F) quantitative analysis revealed the CD3^+^CD8^+^ TILs proportion. (D) Representative flow cytometric analysis and (G) quantitative analysis of PD-1 expression on CD3^+^CD8^+^ TILs. (E) Representative flow cytometric analysis and (H) quantitative analysis revealed CD44^+^CD62L^-^ effector memory T cells (T_em_) proportion gating on CD3^+^CD8^+^ T cells in the spleen tissues. (I) Survival analysis of mice in each group (N = 8). NS: not significant, *: *P* < 0.05, **: *P* < 0.01.

In this study, we found that about 30% of CD3^+^CD8^+^ TILs expressed PD-1 on their surface (Figure 4D and 4H). Consequently, we explored the potential synergistic antitumor effects of combining TP-NLC+L with anti-PD-1 therapy. As illustrated in Figure 5A, TC-1 cells (2 × 10^5^) were subcutaneously injected into C57BL/6 mice to establish the CC tumor model. Seven days post-injection, the mice were randomly divided into six groups: the control, TP-GA-NLC, TP-ICG-NLC+L, TP-NLC+L, anti-PD-1 group, and anti-PD-1+TP-NLC+L groups. TP-NLC administration and laser irradiation were conducted as described previously, while the anti-PD-1 antibody was administered intraperitoneally 24 h post-laser irradiation. Tumor volumes were assessed every two days, revealing a substantial increase in tumor volume in both the control and anti-PD-1 groups. Compared with the control group, the treatment with TP-GA-NLC and TP-ICG-NLC+L resulted in tumor growth inhibition, with TP-NLC+L demonstrating a more pronounced inhibitory effect on tumor growth (Figure 5B). Notably, TP-NLC+L in combination with anti-PD-1 therapy not only significantly inhibited tumor growth, but also resulted in complete tumor regression in two out of six mice.

To further investigate the effects of TP-NLC+L combined with PD-1 blockade on the adaptive antitumor immune response, CD3^+^CD8^+^ TILs and CD8^+^ T_em_ cells in the spleens were analyzed. As shown in Figure 5C and 5F, compared with the control group, the percentage of CD3^+^CD8^+^ TILs was significantly increased in the TP-NLC+L and anti-PD-1+TP-NLC+L groups, but no obvious changes were observed in the TP-GA-NLC, TP-ICG-NLC+L and anti-PD-1 groups.

The anti-PD-1+TP-NLC+L therapy exhibited a slightly higher percentage of CD3^+^CD8^+^ TILs (33.5%) relative to the TP-NLC+L therapy (29.2%) but without statistical significance. Furthermore, a marked down-regulation of PD-1 expression on CD3^+^CD8^+^ TILs was observed in mice treated with anti-PD-1 antibody, but not in those treated with TP-NLC+L (Figure 5D and 5G). The combination of PD-1 blockade with TP-NLC+L also resulted in a slight down-regulation of PD-1 expression on the CD3^+^CD8^+^ TILs, although this change was not statistically significant. These results indicated that PD-1 blockade could act not only by disrupting the PD-1/PD-L1 interaction, but also by down-regulating the expression of PD-1 on the CD3^+^CD8^+^ TILs. Insufficient T cell infiltration into tumor tissues and low expression of PD-L1 might be the potential mechanisms underlying resistance to anti-PD-1/PD-L1 immunotherapy. As shown in Figure 5B and 5G, the treatment with anti-PD-1 alone reduced PD-1 expression on CD3^+^CD8^+^ TILs, but was insufficient to control tumor growth. These results indicated that cervical cancer exhibited resistance to anti-PD-1 therapy, and merely down-regulating the expression of PD-1 did not overcome the anti-PD-1 resistance. As illustrated in Figure 5B and 5F, the combination of TP-NLC+L treatment with anti-PD-1 therapy enhanced the infiltration of CD3^+^CD8^+^ TILs into tumor tissues and significantly inhibited tumor growth. Based on these results, it was suggested that TP-NLC+L might overcome anti-PD-1 resistance by promoting the infiltration of CD3^+^CD8^+^ TILs. Moreover, T_em_ cells in the spleens were also assessed by flow cytometry, and the results showed that the anti-PD-1+TP-NLC+L group exhibited the highest percentage of T_em_ cells, which were 5.06 times greater than those in the control group (Figure 5E and 5H). To further evaluate the immunotherapeutic efficacy of TP-NLC+L combined with PD-1 blockade, the overall survival of the mice was monitored. In the control group, all mice succumbed within 18 days, and no significant prolongation of survival was observed in the anti-PD-1 group, potentially due to inadequate CD8^+^ T cells infiltration in tumor tissues. Notably, survival was significantly prolonged in the TP-NLC+L group. Remarkably, the most pronounced antitumor efficacy was observed in the anti-PD-1+TP-NLC+L group, with 50% of the mice surviving beyond 7 weeks (Figure 5I). These results suggested that, in addition to arresting tumor growth, PD-1 blockade combined with TP-NLC+L therapy elicited a robust systemic antitumor immune response, resulting in the long-term survival of mice with cervical cancer.

### The systemic antitumor immune response of TP-NLC+L combined with PD-1 blockade

**Figure 6:**
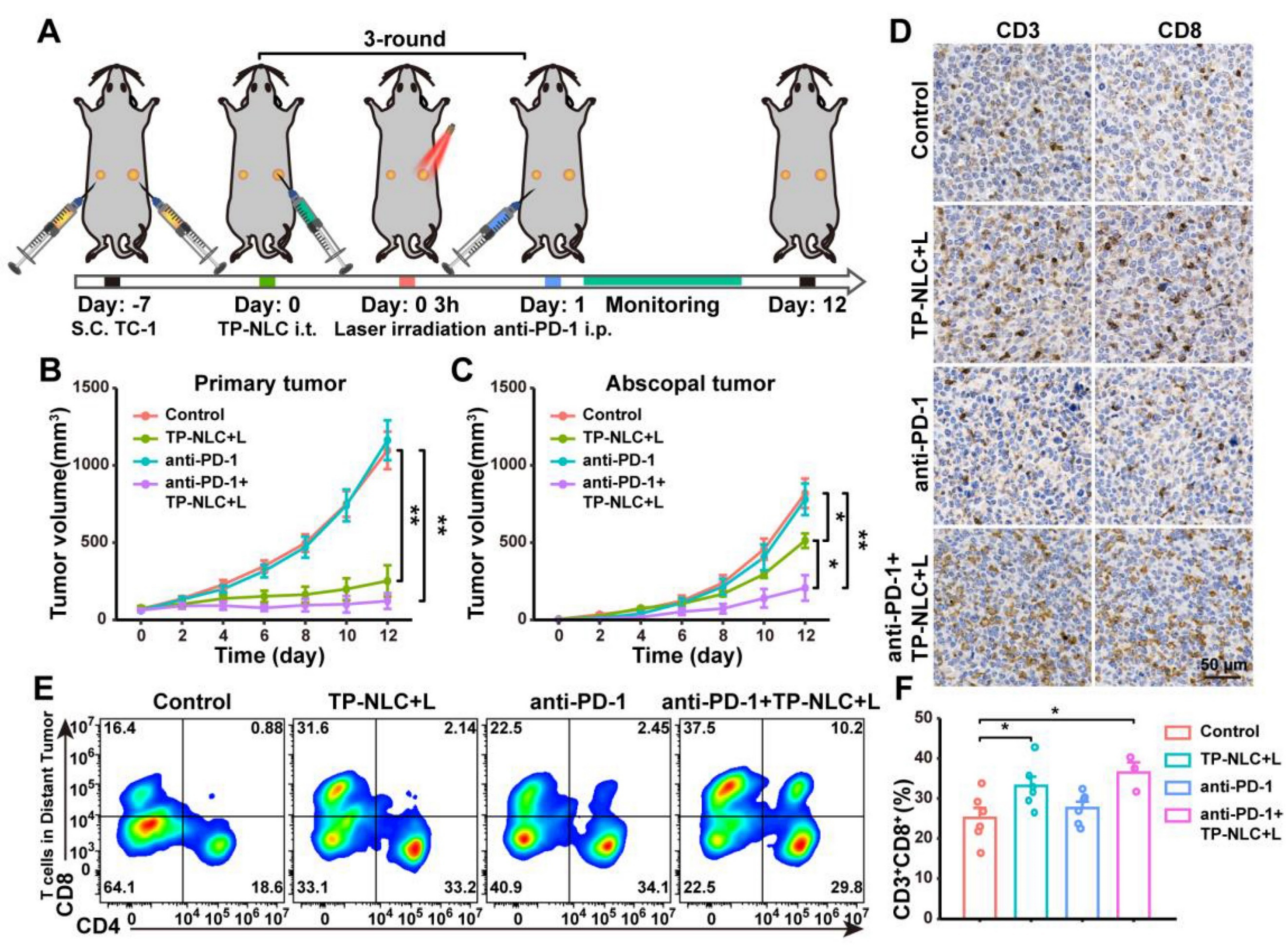
TP-NLC+L+anti-PD-1 therapy inhibited the abscopal tumor growth. (A) Treatment schedule for TP-NLC+L+anti-PD-1 therapy in bilateral TC-1 tumor model. Tumor growth curves of (B) the primary tumors and (C) the abscopal tumors in different groups on day 12. (D) The representative CD3 and CD8 immunohistochemistry staining images of the abscopal tumor tissues. Scale bar: 50 μm. (E) Representative flow cytometric analysis and (F) quantitative analysis of CD8^+^ cells proportion gating on CD3^+^ T cells in the abscopal tumor tissues. NS: not significant, *: *P* < 0.05, **: *P* < 0.01.

To further confirm that PD-1 blockade in combination with TP-NLC+L therapy could elicit a robust systemic antitumor immune response, a bilateral TC-1 tumor-bearing C57BL/6 mouse model was employed. As illustrated in Figure 6A, the bilateral TC-1 tumor model was established by subcutaneously injecting 2 × 10^5^ TC-1 cells into the right flank (designated as primary tumor) and 1 × 10^5^ TC-1 cells into the left flank (designated as abscopal tumor) of the mice. Seven days after TC-1 cells injection, the mice were randomly divided into the control group, the TP-NLC+L group, the anti-PD-1 group, and the anti-PD-1+TP-NLC+L group. TP-NLC was injected into the primary tumors every three days for a total of three times, with laser irradiation conducted 3 h following each TP-NLC injection, and the administration of the anti-PD-1 antibody was carried out as previously described (Figure 6A). The volumes of both primary and abscopal tumors were measured every two days. As shown in Figure 6B and C, the TP-NLC+L treatment demonstrated significant efficacy in inhibiting the growth of both primary and abscopal tumors. The combination of PD-1 blockade with TP-NLC+L demonstrated the most effective control over primary tumors, whereas anti-PD-1 blockade alone was insufficient to inhibit primary tumor growth. Notably, in addition to effectively inhibiting primary tumors, the anti-PD-1+TP-NLC+L treatment also arrested the growth of abscopal tumors, suggesting that the combination therapy elicited a robust systemic antitumor immune response. To further investigate the effects of the antitumor immune response, CD8^+^ TILs in the abscopal tumors were assessed. As shown in Figure 6D, immunohistochemistry staining revealed enhanced recruitment of CD8^+^ TILs to the abscopal tumors following treatment with the combination of PD-1 blockade and TP-NLC+L. Subsequent flow cytometric analysis and quantification showed a significant increase in the percentage of CD3^+^CD8^+^ TILs within the abscopal tumors in the anti-PD-1+TP-NLC+L group, consistent with the immunohistochemistry results (Figure 6E and 6F). Based on these results, the combination of anti-PD-1 with TP-NLC+L therapy could initiate a systemic antitumor immune response that enhanced recruitment of CD8^+^ T cells to the tumor microenvironment and inhibited the growth of distal tumor.

### The biosafe of TP-NLC

Biological safety is essential for the clinical translation of nanomedicines. Therefore, blood samples from mice were taken to examine alanine aminotransferase (ALT), aspartate aminotransferase (AST), UREA, and creatinine (Cr), in order to evaluate liver and kidney function at the experimental endpoint of antitumor effect of TP-NLC+L *in vivo*. As presented in Figure S19, with the exception of one mouse exhibiting elevated ALT levels in the GA+ICG group and another showing increased AST levels in the TP-NLC group, no abnormalities in liver or kidney function were detected among the control, GA, ICG+L, GA+ICG+L, GA/ICG-NLC+L, TP-NLC and TP-NLC+L groups. Additionally, major organs (heart, liver, spleen, lung, and kidney) from the seven groups were isolated and sliced for H&E staining. The histopathological analysis revealed no toxic pathological alterations in the major organs within these groups (Figure S20). These findings collectively suggest that TP-NLC exhibits favorable biocompatibility, supporting its potential for clinical translation.

## Conclusions

In conclusion, we developed a GA/ICG co-loaded nanocarrier with tumor targeting capabilities designed to activate systemic antitumor immune response by inducing pyroptosis of CC cells, boosting the efficacy of PD-1 blockade, and arresting tumor growth. The plant-derived agent GA and the photosensitizer ICG were co-loaded into a TP-NLC nanocarrier, which was further modified with TMTP1 peptide, demonstrating an enhanced capacity to induce pyroptosis. GA exerted a dual role in the TP-NLC+L-induced pyroptosis process: it directly triggered pyroptosis, and enhanced PDT effects by inhibiting the intracellular antioxidant systems. Moreover, the TMTP1 peptide modification improved the targeting efficiency of GA and ICG to tumor tissues. The TP-NLC-based chemotherapy and PDT synergistically triggered pyroptosis, contributing to tumor growth inhibition, DCs maturation, and expansion of CD3^+^CD8^+^ TILs. Further mechanistic studies revealed that TP-NLC+L triggered pyroptosis through a ROS-dependent caspase-3/GSDME pathway. The combination therapy of TP-NLC+L and PD-1 blockade effectively inhibited tumor growth, promoted DCs maturation, initiated the CD3^+^CD8^+^ TILs expansion and recruitment, and enhanced adaptive antitumor efficiency, thus generating systemic antitumor immune response to suppress the abscopal tumors. Consequently, TP-NLC+L-based chemo-PDT presents an attractive therapeutic approach for the treatment of CC and may serve as a promising immunotherapeutic strategy to improve efficacy of PD-1 blockade through the induction of pyroptosis.

## Methods

### Materials

The GA and ICG were purchased from Selleck Chemicals (Shanghai, China). Compritol 888 ATO was obtained from Guangzhou Standard Pharma Co., Ltd. (Guangzhou, China). Medium chain triglycerides (MCT) and 1, 2-distearoyl-sn-glycero-3-phosphoethanolaminepolyethylene glycol-2000 (DSPE-PEG_2000_) were bought from Shanghai YuanYe Biotechnology Co., Ltd. (Shanghai, China). DSPE-PEG_2000_-DBCO was purchased from Xian Ruixi Biological Technology Co., Ltd. (Xian, China). Lecithin was from Avito Pharmaceutical Technology Co., Ltd. (Shanghai, China). Tween-80 was obtained from MedChemExpress (MCE) (New Jersey, USA). N(3)-TMTP1 was supplied through Bankpeptide Biological Technology Co., Ltd. (Hefei, China). Dulbecco's modifed Eagle's medium (DMEM) and Roswell Park Memorial Institute (RPMI) 1640 medium were purchased from GIBCO Invitrogen Corp (Gibco, USA). Fetal bovine serum was obtained from Zhejiang Tianhang Biological Technology Co., Ltd. ATP assay kit, LDH assay kit, DCFH-DA cellular ROS detection assay kit and N-Acetyl-L-cysteine (NAC) were purchased from Biyuntian Biotechnology (Shanghai, China). Tumor Dissociation Kit was obtained from Miltenyi Biotec (Bergisch Gladbach, Germany). Anti-CD3, anti-CD8 and anti-GSDME antibodies were obtained from Abcam Company (Toronto, Canada). Anti-caspase-3 antibody was purchased from Cell Signaling Technology (CST) (MA, USA). Annexin V-FITC/PI apoptosis and necrosis detection kit, APC-Cy7-anti-CD45, BV421-anti-CD3e, BV605-anti-CD4, FITC-anti-CD8a, PE-anti-CD279 (PD-1), PE-Cy7-anti-CD44, APC-anti-CD62L, PerCP-CY5.5-anti-CD11c, V500-anti-I-A/I-E, BV650-anti-CD80, BV786-anti-CD86, anti-CD16/CD32 and percoll were purchased from BD Pharmacia (San Diego, CA). Anti-mouse PD-1 was obtained from BioXCell, Inc. (West Lebanon, USA).

### Cell culture and animals

Human cervical cancer cell line HeLa and human keratinocytes cell line HaCaT were obtained from the American Type Culture Collection (ATCC) and mouse lung epithelial cell line TC-1 with HPV16 E6/E7 was purchased from Tumor Cell Bank of the Chinese Academy of Medical Sciences (Beijing, P.R. China). HeLa, TC-1 and HaCaT cells were cultured in DMEM or 1640 medium supplemented with 10% FBS and 1% penicillin/streptomycin at 37°C under 5% CO_2_, respectively. The female C57BL/6 mice (4-6 weeks old) were obtained from Jicui Yaokang Biological Technology Co., Ltd (Nanjing, China) and maintained in the Animal Experimental Center of Tongji Hospital of Huazhong University of Science and Technology (HUST) under specific pathogen-free (SPF) conditions. The animal protocol was granted by the Ethics Committee of Tongji Hospital, Tongji Medical College, HUST (protocol code: TJH-202211014).

### Preparation of TP-NLC carrier

The GA and ICG co-loaded TP-NLC nanocarrier modified with the TMTP1 peptide was prepared by the emulsification and solvent evaporation method. Briefly, a 10 mL PBS solution containing 300mg of tween-80 was used as the aqueous phase. Lecithin (50 mg), GA (5 mg) and ICG (5 mg) were dissolved in 2 mL ethanol, while Compritol 888 ATO (60 mg), medium chain triglycerides (30 mg) and DSPE-PEG_2000_-DBCO (11 mg) were dissolved in 1 mL chloroform with heating, the two organic solutions were mixed together to form the oil phase. The oil phase was dropped into the aqueous under magnetic stirring at a temperature range of 65 °C to 70 °C. The stirring was continued until the organic solution had completely evaporated, after which the solution was further stirred at 4 ℃ for 2 h. Then, N(3)-TMTP1 was added into the solution and incubated at room temperature for 1 h. Finally, TP-NLC was obtained after removing the unlinked N(3)-TMTP1 peptide with ultrafiltration tube (MWCO: 100 kDa). TMTP1-modified single-drug-loaded nanocarriers TP-ICG-NLC and TP-GA-NLC were prepared using the same protocol as above. For the preparation of the GA/ICG-NLC nanocarrier, DSPE-PEG2000-DBCO was replaced with DSPE-PEG2000, and the N(3)-TMTP1 peptide modification was omitted. TP-Cou-6-NLC and Cou-6-NLC were prepared by replacing the ICG with 500 μg of Cou-6.

### Characteristics of TP-NLC

The particle sizes and zeta potentials of TP-NLC were measured by DLS. The morphology of the nanocarriers was observed and imaged by TEM. The coupling efficiency (CE) of the N(3)-TMTP1 peptide and the encapsulation efficiency (EE) of GA were measured by high-performance liquid chromatography (HPLC). The EE of ICG was measured by UV-vis spectrophotometer.

The calculated formulas for CE of N(3)-TMTP1 and EE were as follows:

CE% = (1-W_free peptide_ / W_peptitde added_) ×100%; EE% = (1-W_free drug_ / W_total drug_) ×100%

In the formula, W_free peptide_ represented the unlinked N(3)-TMTP1 peptide content, W_peptitde added_ refers to the total N(3)-TMTP1 peptide content when preparing the TP-NLC, W_free drug_ represented the drug content in the ultrafiltrate of nanocarriers and W_total drug_ represented the drug content in the nanocrriers including the ultrafiltrate. The HPLC conditions for N(3)-TMTP1 detection were as follows. 25 μL N(3)-TMTP1 peptide solution was assayed by HPLC equipped with a C18 column (4.6×250 mm, 5 μm). The mobile phase was acetonitrile (1% trifluoroacetic acid): water (1% trifluoroacetic acid) (20/80, v/v), at a flow rate of 1.0 mL/min. Detection was conducted at 25 ℃ with detection wavelength 214 nm. For the detection of GA, the sample volume injected was 20 μL and the mobile phase was acetonitrile (1% trifluoroacetic acid): water (1% trifluoroacetic acid) (90/10, v/v) with 1.0 mL/min flow rate. UV absorbance was monitored at a wavelength of 361 nm, and the column temperature was 30 °C. ICG was dissolved in DMSO and measured on UV-visible spectrophotometer at a wavelength of 780 nm.

The stabilities of TP-NLC and GA/ICG-NLC in PBS and 10% FBS at 37℃ were assessed by measuring the size of the nanocarriers using DLS within 96 h.

The photobleaching experiments were conducted as follows: the same ICG concentration (100 µg/mL) of free ICG, TP-ICG-NLC and TP-NLC was placed into transparent polypropylene tube and exposed to 808 nm NIR light for 5 min at 2 W/cm^2^. The color change of solutions were observed and imaged.

The free ICG (18.5 µg/mL) and the nanocarriers of TP-ICG-NLC and TP-NLC (with an equivalent concentration of 12.0 µg/mL of free ICG) were placed into transparent polypropylene tube and exposed to 808 nm NIR light for 10 min at 1.2 W/cm^2^. UV-Vis absorption spectra were measured every 2 min during NIR exposure.

The photothermal stability of TP-NLC carrier was measured by turning on/off the laser for five cycles. The same ICG concentration (100 µg/mL) of free ICG, TP-ICG-NLC and TP-NLC was placed into transparent polypropylene tube and exposed to 808 nm NIR light (2 W/cm^2^) for 3 min, followed by a cooling period to room temperature with the laser turned off. Temperature variations were recorded per 30 seconds.

The release profiles of GA and ICG from GA/ICG-NLC and TP-NLC were evaluated by the dialysis method. In brief, GA, ICG, GA/ICG-NLC, and TP-NLC solution (2 mL) were separately placed into a dialysis bag (MWCO 8000-140,000), which was immersed into 200 mL of 0.5% SDS phosphate buffer at 37 °C for 48 h with magnetic stirring (100 rpm). 1 mL sample was taken out at 1 h, 2 h, 4 h, 24 h, and 48 h, and the same volume of 0.5% SDS phosphate buffer were added. Then, ethyl acetate was added to extract GA. The concentrations of GA and ICG were measured by HPLC and UV-visible spectrophotometer analysis, respectively.

### Hemolysis assay

Briefly, 500 μL of 2% red blood cell (RBC) suspension was individually incubated with NLC (without GA and ICG loading), GA/ICG-NLC and TP-NLC with different ICG concentrations (1, 2, 5, 10, 20, 50, and 100 µg/mL) and GA concentrations (0.02, 0.04, 0.1, 0.2, 0.4, 1, and 2 µg/mL) at 37 °C for 6 h. Additionally, 0.1% Triton X-100 was regarded as the positive control, whereas PBS was the negative control. After incubation, the mixtures were centrifuged with 3500 rpm for 10 min, and measured the optical absorbance (OD) at 570 nm with a microplate reader. The hemolysis ratio was calculated as follows: hemolysis ratio (%) = (OD value of sample - OD value of negative control) / (OD value of positive control - OD value of PBS) × 100%.

### Cellular uptake of TP-NLC *in vitro*

HeLa, TC-1 and HaCaT cells were cultured onto sterilized glass slides in 12-well plates (5 × 10^5^ cells per well) for 24 h, and the medium was replaced with fresh medium containing TP-Cou-6-NLC, Cou-6-NLC, and free Cou-6 with an equivalent concentration for 1 h. The TP-Cou-6-NLC Blocking group was pre-treated with 10 μM TMTP1 cyclic peptide as competitive inhibitor for 10 min prior to adding of the TP-Cou-6-NLC. Then, the cells washed three times and fixed with 4% paraformaldehyde. Afterward, the cell nuclei were stained with 4',6-diamidino-2-phenylindole (DAPI) and observed by confocal laser microscopy.

### Biodistribution of TP-NLC carrier *in vivo*

To establish a subcutaneous cervical cancer model, TC-1 cells (2×10^5^) were inoculated subcutaneously into C57BL/6 mice. When the tumor volumes reached 150 mm^3^, the mice were randomly divided into the free ICG, NLC, TP-NLC, and Blocking (TMTP1+TP-NLC) groups (n = 6 in each group). In each group, the mice were given injection of the corresponding nanocarriers via the tail vein (free ICG: 5 mg/kg, NLC and TP-NLC: dose equivalent to 5 mg/kg dose of free ICG). In the Blocking group, a cyclic peptide (35 μM TMTP1) was pre-injected as a competitive inhibitor 10 minutes prior to the administration of TP-NLC. At 2 h, 4 h, 8 h, 12 h, and 24 h after post-injection, the mice were anesthetized with isoflurane and monitored by an IVIS Spectrum Imaging System (PerkinElmer, USA, excitation wavelength: 745 nm, emission wavelength: 840 nm). At 4 h and 24 h post-injection, three mice from each group were randomly sacrificed, and their tumor tissues, along with major organs (heart, liver, spleen, lung, and kidney), were isolated for NIR fluorescence imaging. The region of interest (ROI) tool in the self-contained software system (Living Image® software 4.0) was applied to quantify the fluorescence signal intensity.

### *In vitro* LDH release assay

HeLa and TC-1 cells were cultured in 12-well plates (5 × 10^5^ cells per well) for 24 h and divided into seven groups: the control group, the free ICG+L group, the GA group, the GA+ICG+L group, the GA/ICG-NLC+L group, the TP-NLC group, and the TP-NLC+L group (+L, with laser irradiation). In accordance with the group assignments, different drug formulations were added to the corresponding wells (GA concentration: 0.8 μg/mL; ICG concentration: 40 μg/mL). Then, the medium was replaced by fresh medium after 3 h, the ICG+L, GA+ICG+L, GA/ICG-NLC+L and TP-NLC+L groups received 808 nm laser irradiation (0.5 W/cm^2^) for 5 min. After 21 h further incubation, 120 μL of cell culture supernatant were collected and added into 96-well plate containing 60 μL of LDH detection reagents (Beyotime Biotechnology, C0017). The solutions were mixed and incubated for 30 min at room temperature, shield from light exposure. The fluorescence of the mixed solutions at 490 nm was measured by a microplate reader (SpectraMax ABS Plus).

### *In vitro* intracellular ATP detection

Cell culture and subsequent treatment were conducted as outlined in the preceding section. Following the treatment, the cells were further cultivated for 21 h. Then, the cells were lysed and subjected to centrifugation to obtain the cell lysate. According to the operating manuals of ATP Assay Kit (Beyotime Biotechnology, China), 100 μL of ATP detection reagent was added into black 96-well plates and allowed to equilibrate for 5 min. Thereafter, 20 μL of cell lysate was added and mixed quickly. The luminescence signal of the samples was measured by a Synergy 2 multifunction microplate reader to assess ATP levels.

### ROS generation assay in extracellular levels

Extracellular ROS *in vitro* was detected using a DCFH-DA probe. The DCFH-DA probe was diluted and subsequently mixed with 0.01 M sodium hydroxide (NaOH), followed by incubation for 30 minutes at room temperature. According to the experimental design, the control group consisted of blank control (DCFH-DA+L) and negative control (DCFH-DA+ICG), the experimental group included the ICG+L group (DCFH-DA+ICG+L), the GA+ICG+L group (DCFH-DA+GA+ICG+L), the GA/ICG-NLC+L group (DCFH-DA+ GA/ICG-NLC +L), and the TP-NLC+L group (DCFH-DA+ TP-NLC +L) (+L, with laser irradiation). Then, the corresponding nanocarriers were added into the mixture containing the DCFH-DA probe. After a 10 min incubation period, the mixture was subjected to 808 nm laser irradiation at an intensity of 0.5 W/cm² for 5 minutes. The fluorescence was then measured using a microplate reader with an excitation wavelength of 485 nm and an emission wavelength of 530 nm.

### Annexin V-FITC/PI assay

Cell culture and treatment were conducted as described in the LDH release assay section. Briefly, after treatment and culture, HeLa or TC-1 cells were digested and collected by centrifugation for Annexin V-FITC/PI staining (BD Bioscience, USA). The stained cells were monitored with flow cytometer (Beckman-Coulter Inc, USA).

### *In vitro* intracellular ROS detection

Intracellular ROS *in vitro* was detected utilizing a DCFH-DA probe. Cell culture and treatment were performed as described in the LDH release assay section. For flow cytometric analysis: HeLa or TC-1 cells were harvested post-treatment via centrifugation and subsequently resuspended in diluted DCFH-DA probe. The cell-probe mixture was incubated at 37 °C for 20 minutes, with mixing every 5 minutes, while being protected from light. After incubation, the cells washed and resuspended in a serum-free medium for flow cytometric analysis. For fluorescence microscopy: DCFH-DA working solution was added into the 12-well plates, which were kept in the dark and incubated at 37 °C for 30 min. The cells were then washed with PBS, treated with an anti-fluorescence quencher, and monitored and photographed under a fluorescence microscope (Olympus, Japan).

### Western blot assay

The expressions of GSDME and caspase-3 in HeLa and TC-1 cells were detected by western blot analysis. Total protein was extracted from cultured cells and tumor tissues by lysing them with RIPA lysis buffer. After denaturation, proteins were separated by 10% SDS-PAGE and then transferred onto the polyvinylidene difluoride (PVDF) membranes. The membranes were then blocked with 5% bovine serum albumin (BSA) for 1 h. Subsequently, the membranes were incubated with primary antibodies at 4 ℃ overnight, followed by incubation with the corresponding secondary antibodies at room temperature for 1 h. Finally, immunoreactive bands were detected by an enhanced chemiluminescence (ECL) kit (Advansta) and visualized with a multimode chemiluminescence system (Bio-Rad, USA). The GAPDH protein was employed as an endogenous control.

### Mitochondrial membrane potential assay

Cell culture and treatment procedures were conducted in accordance with the methodology outlined in the LDH release assay. After treatment, the cells were co-incubated with JC-1 staining solution for 30 min, and the changes of mitochondrial membrane potential were observed by fluorescence microscopy in the green channel (J-monomers) and the red channel (J-aggregates), respectively.

### *In vivo* antitumor efficacy of TP-NLC+L

To establish the CC subcutaneous xenograft tumor model, 2 × 10^5^ TC-1 cells were subcutaneously injected into the right flank of C57BL/6 mice. Seven days later, all the mice were randomly divided into seven groups (n = 6; control, GA, ICG+L, GA+ICG+L, GA/ICG-NLC+L, TP-NLC, and TP-NLC+L), and were injected with PBS, GA, ICG+L, GA+ICG+L, GA/ICG-NLC+L, and TP-NLC via the tail vein once every three days for three times, with GA and ICG dosed at 5 mg/kg. In the ICG+L group, GA+ICG+L group, GA/ICG-NLC+L group, and TP-NLC+L group, tumors were subjected to laser irradiation (808 nm, 0.5 W/cm^2^, 10 min) 4 h post-injection. Tumor volumes and body weights were recorded every two days. The tumor volume was calculated as follows: tumor volume (mm^3^) = width^2^ × length × 0.5. The initial day of administration was designated as day 0. On day 12, all mice were euthanized, and tumors, TDLN, spleens, and the major organs (heart, liver, lung, and kidney) were isolated. The tumor tissues were weighed and photographed. Tumor, TDLN, and spleen tissues were used for the immunocytological analysis, while the remaining tumor tissues were lysed for western blot analysis and sliced for H&E and Ki-67 staining, respectively.

### Antitumor immune response analysis

To assess the antitumor immune responses, fresh tumor tissues, TDLN, and spleen tissues were collected for immunocytes analysis via flow cytometry. Briefly, the tumor tissues were digested using Mouse Tumor Dissociation Kits according to the operating instruction, and then the cell suspensions were filtered with a filter screen (200 mesh, 70 μm). Subsequently, red blood cell lysis buffer was added to remove red cells. The cells were then resuspended in a 40% percoll solution and carefully layered beneath an 80% percoll solution. Following centrifugation at 524 g for 35 minutes, the cells were resuspended in PBS. Then, the cells were stained with Fixable Viability Stain 700 (BD Biosciences, USA) to distinguish between live and dead cells, and anti-CD16/32 antibody was added to block the nonspecific Fc receptor. The relevant antibodies were then added, and the samples were incubated at room temperature for 15 min while protected from light. After incubation, the cells were washed with PBS to remove the excess antibodies and resuspended in a cell staining buffer. Finally, the cells were filtered with a 200-mesh cell strainer and subjected to flow cytometry analysis. Lymph nodes or spleen tissues were ground and filtered with a 200-mesh cell strainer to obtain single-cell suspensions. For detection of mature DCs, the single-cell suspension of TDLNs was stained with V500 anti-mouse I-A/I-E, PerCP-CY5.5 anti-mouse CD11c, BV650 anti-mouse CD80, and BV786 anti-mouse CD86. For detection of memory T cells, the single-cell suspension of spleen tissues was stained with APC-Cy7 anti-mouse CD45, BV421 anti-mouse CD3e, BV605 anti-mouse CD4, FITC anti-mouse CD8a, PE-Cy7 anti-mouse CD44, APC anti-mouse CD62L. Additionally, the expressions of CD3 and CD8 in the abscopal tumors were observed by immunohistochemistry staining to further evaluate the systemic antitumor immune response.

### Combination of TP-NLC+L with PD-1 blockade therapy and abscopal effects in the mice model

A subcutaneous xenograft tumor model of CC was established in C57BL/6 mice, adhering to the protocol described in Section 4.15. All the mice were randomly divided into six groups (n = 6): the control group, the TP-GA-NLC group, the TP-ICG-NLC+L group, the TP-NLC+L group, the anti-PD-1 group, and the anti-PD-1+TP-NLC+L group. The corresponding drugs were administered in each group via tail vein once every three days for three times (the nanocarrier formulations containing GA: 5 mg/kg and ICG: 5 mg/kg). In the TP-ICG-NLC+L group, TP-NLC+L group, and anti-PD-1+TP-NLC+L group, the mice received laser irradiation (808 nm, 0.5 W/cm^2^, 10 min) 4 h after injections, and the mice in the anti-PD-1 group and anti-PD-1+TP-NLC+L groups received intraperitoneal injections of anti-PD-1 antibody at a dose of 200 µg the following day. Tumor volumes were recorded every two days. On day 12, all mice were euthanized, and tumor, TDLN, and spleen tissues were isolated for antitumor immune response analysis, following the protocol specified in Section 4.16.

The subcutaneous xenograft tumor model of CC in C57BL/6 mice was established, and all the mice were randomly divided into four groups (n = 8): the control group, the TP-NLC+L group, the anti-PD-1 group, and the anti-PD-1+TP-NLC+L group. The protocol for administering the drug was consistent with that used in the experiments above. When the maximum tumor diameter exceeded 20 mm or the tumor volume reached 2000 mm^3^, the mice were euthanized and recorded as dead. Seven weeks following the initial treatment, the overall survival rates of the mice were calculated by the Kaplan-Meier approach.

To evaluate the antitumor efficacy of the combination of TP-NLC+L with PD-1 blockade therapy on abscopal tumors, C57BL/6 mice were subcutaneously injected with 2 × 10^5^ TC-1 cells into the right flank (primary tumors) and 1 × 10^5^ TC-1 cells into the left flank (abscopal tumors) to establish bilateral subcutaneous model. Seven days post-inoculation, all the mice were randomly divided into four groups (n = 6): the control group, the TP-NLC+L group, the anti-PD-1 group, and the anti-PD-1+TP-NLC+L group. In the TP-NLC+L and anti-PD-1+TP-NLC+L groups, TP-NLC (with the designated nanocarrier formulations GA at 5 mg/kg and ICG at 5 mg/kg) was injected into the primary tumors once every three days for three times. At 3 h after injection, the primary tumors were exposed to 808 nm laser irradiation (0.5 W/cm^2^) for 10 min. Subsequently, the mice in the anti-PD-1 and anti-PD-1+TP-NLC+L groups received intraperitoneal injections of anti-PD-1 antibody at a dose of 200 µg the following day. Tumor volumes were recorded every two days. On day 12, all mice were euthanized, and the primary tumor tissues were isolated to analyze the expression of CD3 and CD8, following the methodology outlined in Section 4.16.

### Biosafety evaluation

To investigate the biosafety of TP-NLC, at the endpoint of *in vivo* antitumor efficacy of TP-NLC+L experiment, peripheral blood and major organs (heart, liver, spleen, lung, and kidney) were collected from mice in each group. Biochemical indicators in the peripheral blood, including alanine aminotransferase (ALT), aspartate aminotransferase (AST), urea (UREA), and creatinine (CR), were measured by an automated blood biochemical analyzer. The major organs were sliced and stained with H&E for histopathological analysis.

### Statistical analysis

Statistical analyses were performed within the R software environment (version 4.1.2, R Core Team), using the “ggpubr” and “DescTools” R package. All the data have been presented as mean ± standard deviation. For normally distributed data, the differences between the two groups were compared using Student t-test, and the Welch's correction was performed when variances were not equal. For not-normally distributed data, comparisons between two groups were analyzed by Mann-Whitney U test. A p-value of less than 0.05 was considered indicative of statistical significance.

## Supplementary Material

Supplementary figures and tables.

## Abbreviations

ALT: alanine aminotransferase
AST: aspartate aminotransferase
ATP: adenosine triphosphate
CC: cervical cancer
CIN: cervical intraepithelial neoplasia
Cr: creatinine
NAC: N-acetylcysteine
DBCO: dibenzocycloctyne
DCs: dendritic cells
DCFH-DA: 2'7'-dichlorofluorescein diacetate
DLS: dynamic light scattering
DSPE-PEG_2000_-DBCO: 1, 2-distearoyl-sn-glycero-3-phosphoethanolamine (DSPE)-polyethylene glycol 2000 (PEG_2000_)- dibenzocycloctyne (DBCO)
EE: encapsulation efficiency
EPR: enhanced permeability and retention
FBS: fetal bovine serum
FITC: Fluorescein isothiocyanate
GA: gambogic acid
GSH: glutathione
GSDM: gasdermin
GSDMD: gasdermin D
GSDME: gasdermin E
GSDME-N: N-terminal fragment of GSDME
GSDME-FL: full-length GSDME
HE: hematoxylin-eosin
ICB: immune checkpoint blockade
ICG: indocyanine green
LDH: lactate dehydrogenase
MCT: medium chain triglycerides
N(3): azido
NLC: nanostructured lipid carrier
NIR: near-infrared
OS: overall survival
PCD: programmed cell deaths
PD-1: programmed cell death protein 1
PDI: polydispersity
PDT: photodynamic therapy
PEG: polyethylene glycol
PI: propidium iodide
PTT: photothermal therapy
ROS: reactive oxygen species
TAAs: tumor-associated antigens
T_cm_: central memory T cells
T_em_: effector memory T cells
TDLN: tumor-draining lymph nodes
TEM: transmission electron microscopy
TILs: tumor-infiltrating lymphocytes
TP-NLC: TMTP1-modified nanostructured lipid carrier
Trx: thioredoxin
UV-vis: ultraviolet-visible
VEGF: vascular endothelial growth factor
