# Supplementary material for: TMTP1-modified nanocarrier boosts cervical cancer immunotherapy by eliciting pyroptosis
Source: Theranostics. 2025 Apr 13;15(11):5420–39. doi: 10.7150/thno.108357 (PMC12036869; doi:10.7150/thno.108357)
Supplement: Supplementary file 1 — Supplementary figures and tables. [file thnov15p5420s1.pdf]

Supporting Information

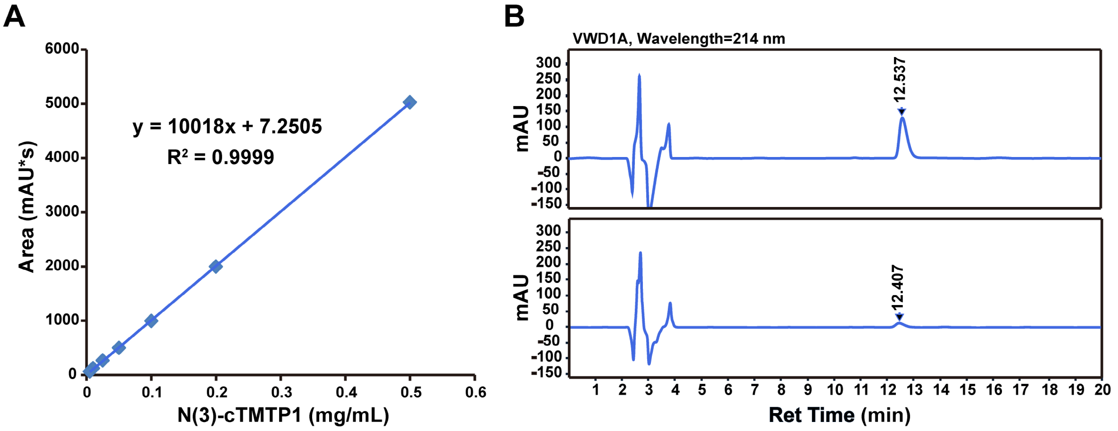

Figure S1. The coupling efficiency analysis of N(3)-TMTP1 peptide. (A) The standard curve of N(3)-TMTP1 peptide was established by the HPLC method. (B) HPLC chromatogram of N(3)-TMTP1 peptide. Upper panel: initial solution of N(3)-TMTP1 peptide, lower panel: ultrafiltrate of N(3)-TMTP1 peptide after the click chemistry reaction.

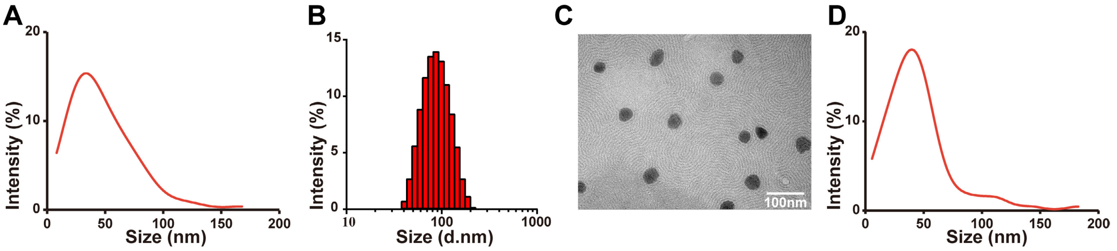

Figure S2. The particle size and morphology of TP-NLC and GA/ICG-NLC nanocarriers. (A) The TP-NLC size distribution of TEM morphology. (B) Hydrodynamic size distribution of GA/ICG-NLC nanocarrier. (C) TEM image and (D) size distribution of GA/ICG-NLC nanocarrier. Scale bar: 100 nm.

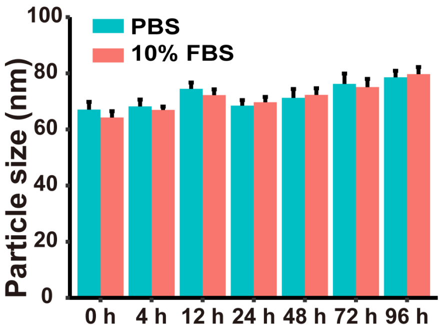

Figure S3. The particle size of GA/ICG-NLC in PBS and 10% FBS within 96 h stored at 37 ℃.

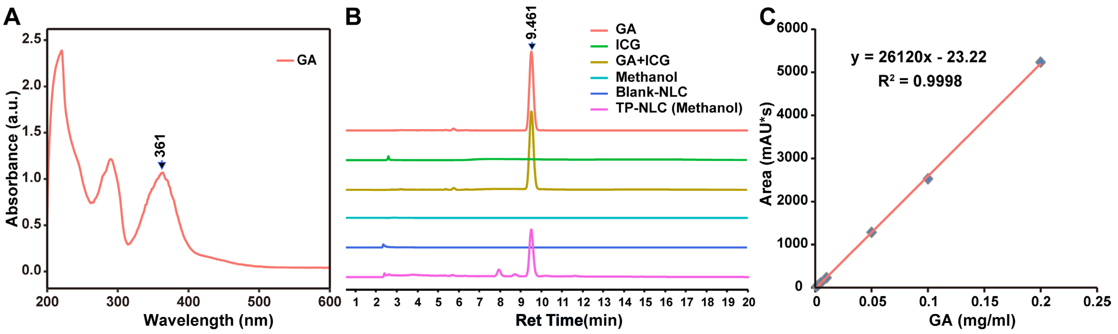

Figure S4. The detection specificity and standard curve of GA. (A) The UV–vis absorption spectra of GA. Black arrow indicated the characteristic absorption peaks. (B) HPLC chromatogram of GA, ICG, GA+ICG, methanol, Blank-NLC (without GA and ICG), and TP-NLC (Methanol). TP-NLC (Methanol): the demulsification of the TP-NLC. (C) The standard curve of GA was established by the HPLC method.

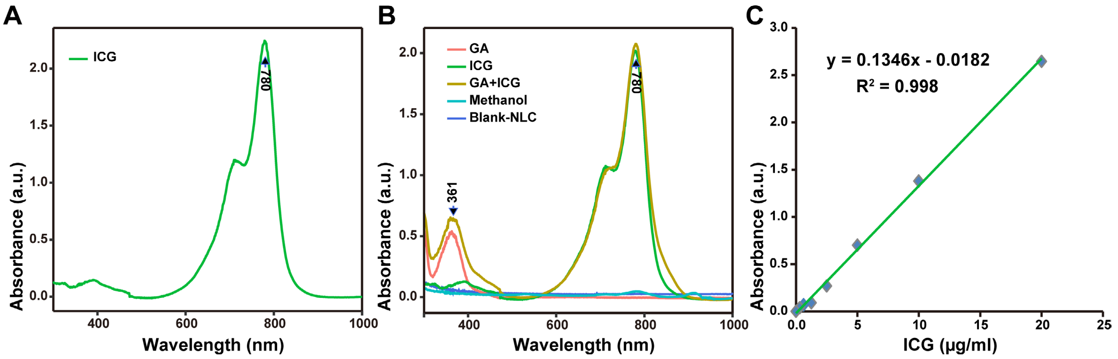

Figure S5. The detection specificity and standard curve of ICG. (A) The UV–vis absorption spectra of ICG. Black arrow indicated the characteristic absorption peaks. (B) The UV–vis absorption spectra of GA, ICG, GA+ICG, methanol, and Blank-NLC (without GA and ICG). (C) The standard curve of ICG was established by the UV-vis method.

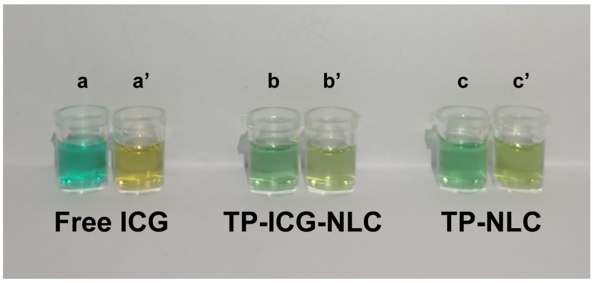

Figure S6. The photograph of free ICG, TP-ICG-NLC, and TP-NLC irradiated with laser irradiation (808 nm, 2 W/cm^2) for 10 min. “a, b, c” represented no laser irradiation treatment, and “a’, b’, c’” represented received laser irradiation.

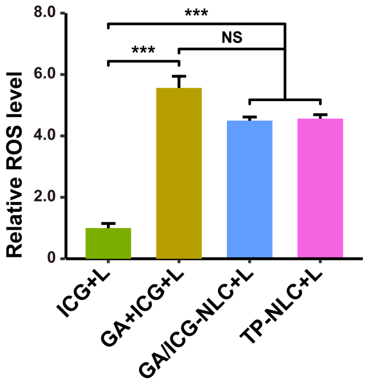

Figure S7. The relative ROS of free ICG, GA+ICG, GA/ICG-NLC，and TP-NLC irradiated with laser irradiation (808 nm, 0.5 W/cm^2) for 5 min. NS: not significant, ***: $P < 0.001$.

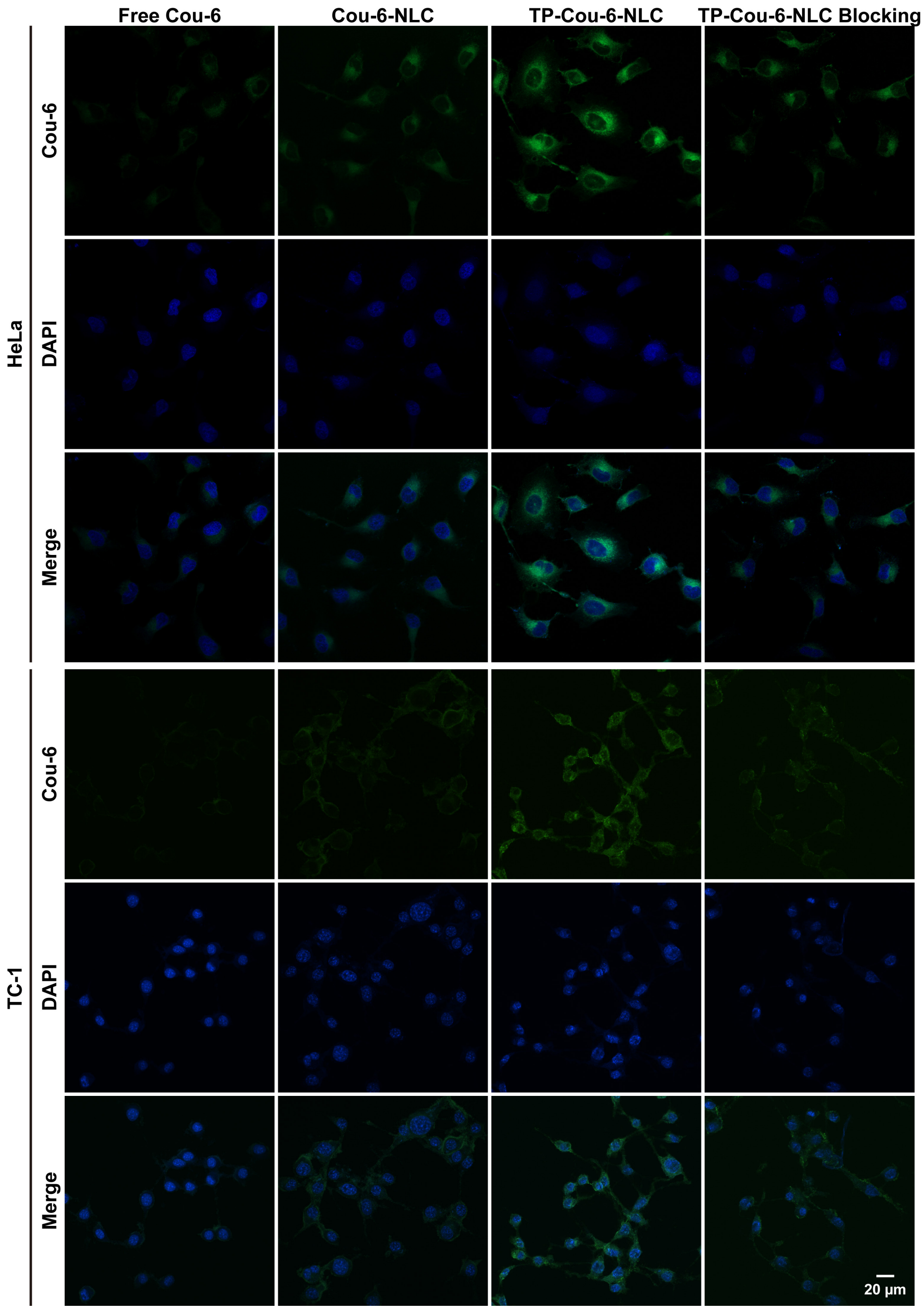

Figure S8. In vitro cellular uptake of free Cou-6, Cou-6-NLC, and TP-Cou-6-NLC in HeLa and TC-1 cells. The green was for Cou-6 and blue was for DAPI. Scale bar: 20 μm.

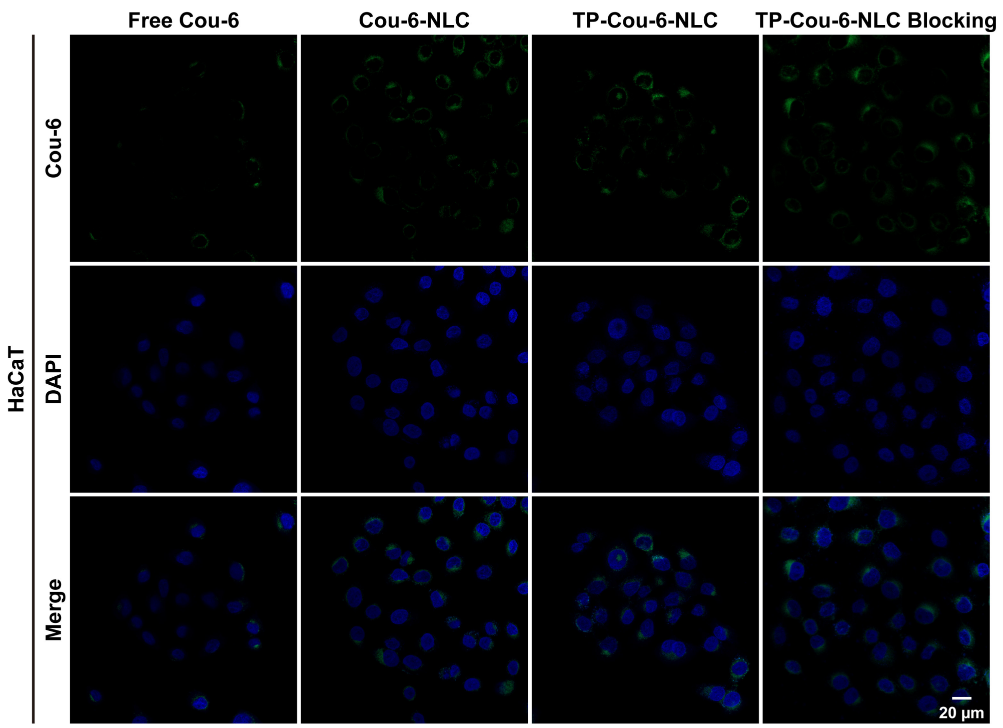

Figure S9. In vitro cellular uptake of free Cou-6, Cou-6-NLC, and TP-Cou-6-NLC in normal HaCaT cells. The green was for Cou-6 and blue was for DAPI. Scale bar: 20 μm.

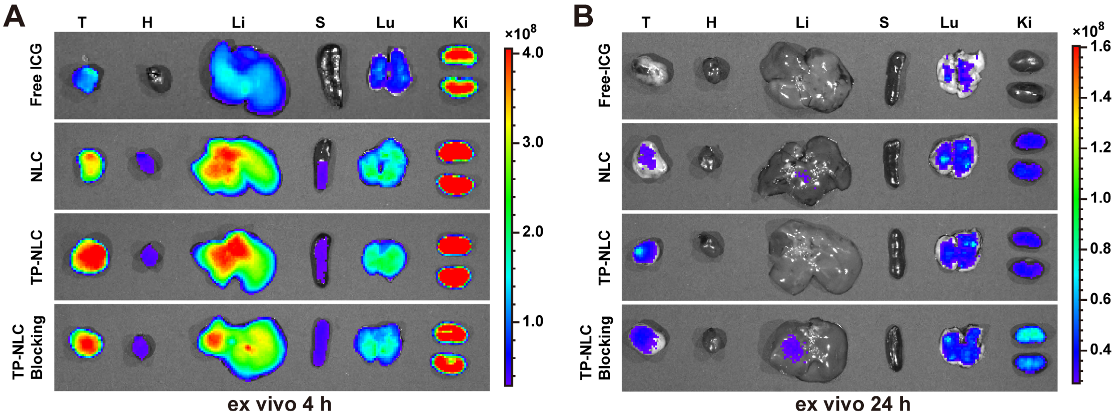

Figure S10. The representative NIR fluorescence images of ex vivo tumor tissues and major organs at (A) 4 h and (B) 24 h after intravenous administration. T: tumor, H: heart, Li: liver, S: spleen, Lu: lung, Ki: kidney. In the TP-NLC Blocking group, TMTP1 peptide as competitive inhibitor was pre-injected by tail vein before the administration of TP-NLC.

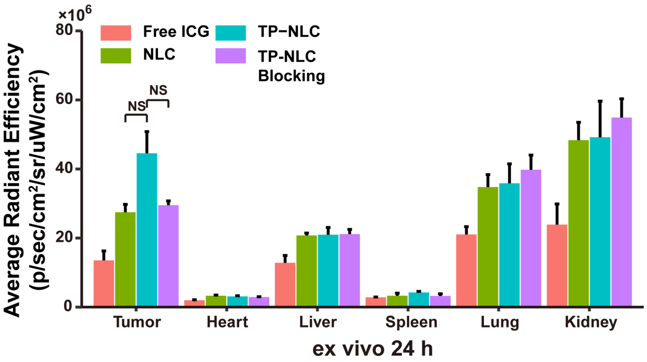

Figure S11. The quantitative analysis of the fluorescence intensity in the tumor, heart, liver, spleen, lung, and kidney tissues at 24 h after intravenous administration.

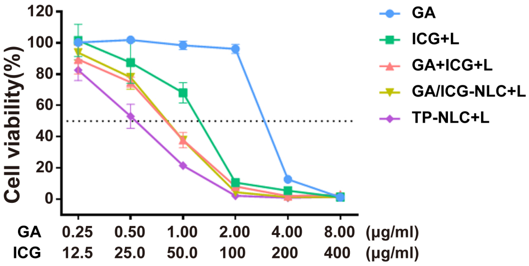

Figure S12. The cell viability of HeLa cells treated with different drugs (GA, ICG+L, GA+ICG+L, GA/ICG-NLC+L, and TP-NLC+L) for 24 h. +L: laser irradiation at 808 nm (0.5 W/cm^2, 5 min).

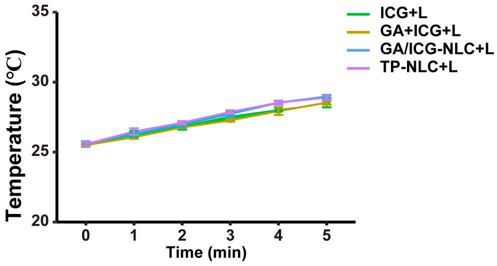

Figure S13. The temperature changes of different drugs (ICG+L, GA+ICG+L, GA/ICG-NLC+L, and TP-NLC+L) for 5 min. +L: laser irradiation at 808 nm (0.5 W/cm^2, 5 min).

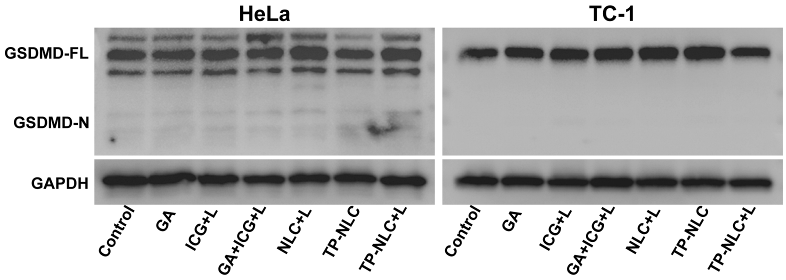

Figure S14. Western blot detection of full-length GSDMD (GSDMD-FL) and GSDMD-N terminal domain (GSDMD-N) expressions in HeLa and TC-1 cells after different treatments.

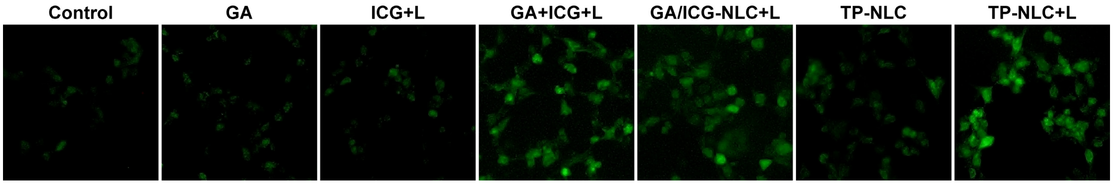

Figure S15. Representative fluorescent images of ROS generation in TC-1 cells after different treatments, detected by the fluorescent dye DCFH-DA. +L: laser irradiation at 808 nm (0.5 W/cm^2, 5 min). Scale bar: 30 μm.

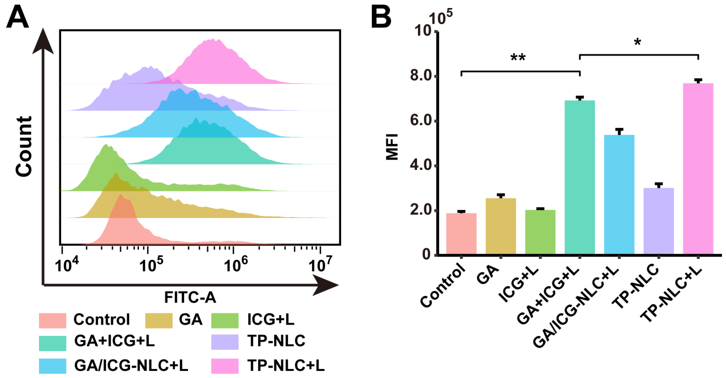

Figure S16. Flow cytometric analysis of ROS generation in TC-1 cells after different treatments, detected by the fluorescent dye DCFH-DA.

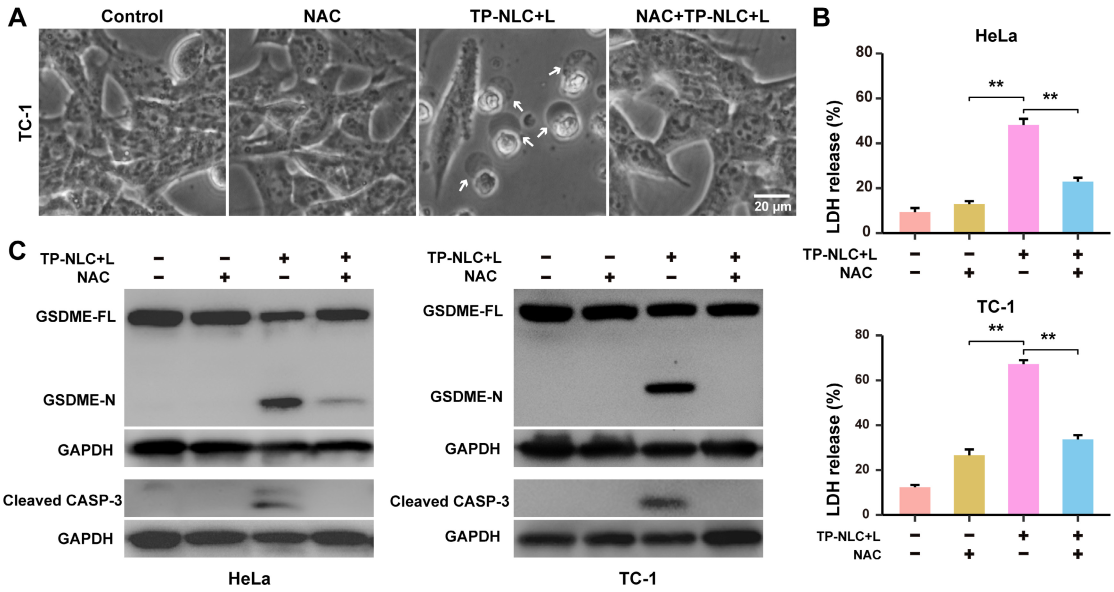

Figure S17. ROS generation was essential for TP-NLC+L-mediated pyroptosis. (A) Representative bright-field images of TC-1 cells treated with TP-NLC+L in the presence or absence of NAC. The white arrows indicated pyroptotic cells. Scale bar: 20 μm. (B) The release of LDH cell viability after different treatments. (C) Western blot detection of full-length GSDME (GSDME-FL), GSDME-N terminal domain (GSDME-N), and Cleaved CASP-3 expressions in HeLa and TC-1 cells after different treatments.

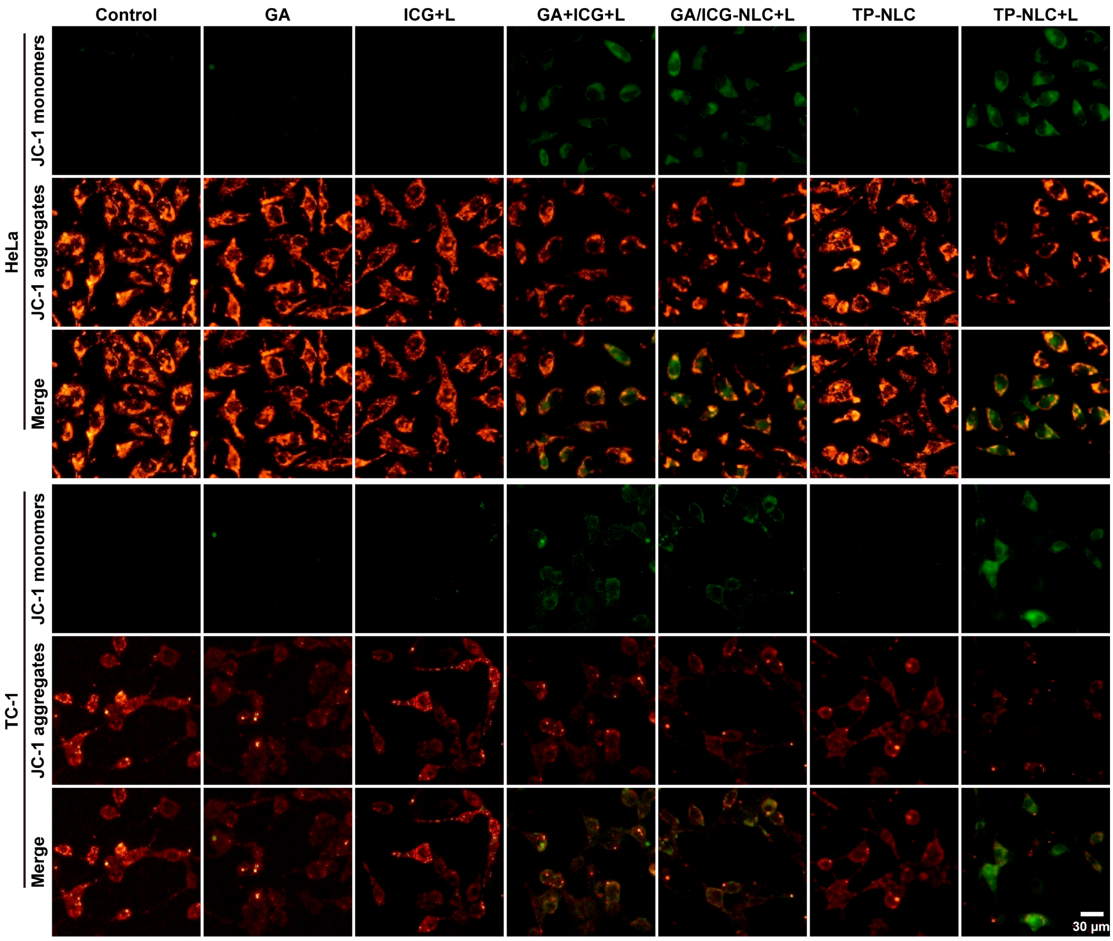

Figure S18. Representative fluorescent images of JC-1 staining in HeLa and TC-1 cells after different treatments (PBS, free ICG+L, GA, GA+ICG+L, GA/ICG-NLC+L, TP-NLC, and TP-NLC+L). +L: laser irradiation at 808 nm (0.5 W/cm^2, 5 min). Scale bar: 30 μm.

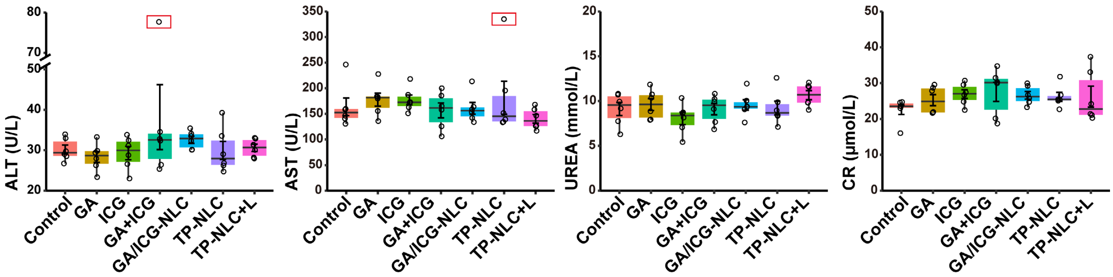

Figure S19. Liver and kidney function indexes including ALT, AST, UREA and CR at the endpoint of the observation. Abnormal high values were highlight with a red frame.

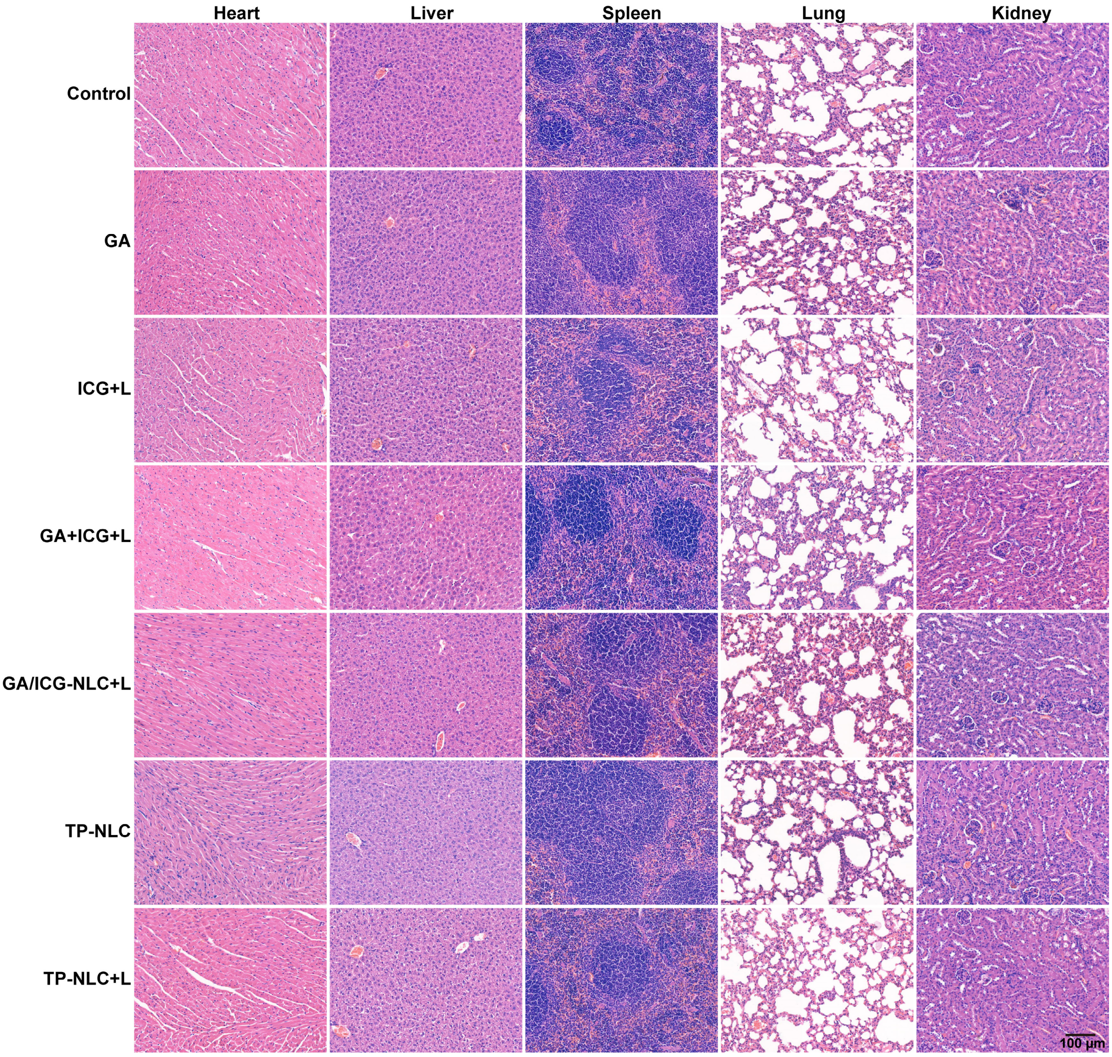

Figure S20. HE staining analysis of major organs (heart, liver, spleen, lung, kidney) in the Control, GA, ICG+L, GA+ICG+L, GA/ICG-NLC+L, TP-NLC, and TP-NLC+L groups.
